# Not just old wine in new bottles: Polygenic liability for ADHD is associated with electrophysiological affective-motivational processing beyond anxiety, depression, and ODD

**DOI:** 10.1038/s41398-025-03434-z

**Published:** 2025-06-24

**Authors:** Kristóf Ágrez, Zsombor Visky, György Hámori, Mária Takács, Attila J. Pulay, János M. Réthelyi, Nóra Bunford

**Affiliations:** 1 HUN-REN Research Centre for Natural Sciences, Clinical and Developmental Neuropsychology Research Group, Budapest, Hungary; 2 Semmelweis University, Faculty of Medicine, Budapest, Hungary; 3 Budapest University of Technology and Economics, Department of Cognitive Science, Budapest, Hungary; 4 Semmelweis University, Faculty of Medicine, Department of Psychiatry and Psychotherapy, Budapest, Hungary; 5 Doctoral School of Semmelweis University, Mental Health Sciences Division, Budapest, Hungary

**Keywords:** Clinical genetics, Neuroscience, ADHD

## Abstract

In attention-deficit/hyperactivity disorder (ADHD), emotional features account for heterogeneity and exacerbate severity of behavioral and functional impairments, beyond cognitive and comorbidity features. Yet, debate remains about the extent to which, in ADHD, such emotional features are a “core feature”, i.e. whether ADHD should be conceptualized as encompassing difficulties with regulating not only activity, attention, and impulses but also processing and regulating emotions. We aimed to address this issue by examining the extent to which in adolescents, ADHD polygenic scores (PGSs) are associated with electrophysiological indices of affective-motivational processing, measured during a monetary punishment/reward feedback paradigm. ADHD PGSs were negatively associated, in *n* = 166 adolescents (*M*_age_ = 15.76 years, *SD* = 1.07; 42.77% girls), with amplitude values of an occipitoparietal event-related potential (i.e. late positive potential) and were positively associated, in *n* = 84 adolescents (*M*_age_ = 15.76 years, *SD* = 1.05; 41.67% girls), with fronto-centro-parietal alpha event-related desynchronization. Across analyses, covariates were anxiety, depression, and ADHD with comorbid disruptive behavior disorder PGSs; ADHD, internalizing, and oppositional defiant disorder severity; childhood maltreatment; current ADHD medication; and baseline values of the outcome. Findings were replicated in sensitivity analyses with blocks of conceptually related covariates entered separately. In adolescents, electrophysiological indices of affective-motivational processing are associated principally with genetic liability for ADHD but not comorbidity genetic liability or comorbidity manifest symptoms.

## Introduction

Attention-deficit/hyperactivity disorder (ADHD) is a mechanistically and phenotypically heterogeneous disorder and this heterogeneity has hindered progress in determining etiology and identifying precise predictors of prognosis [1, 2]. In attempts to disentangle this heterogeneity, the field has been focusing on characteristics along which the ADHD clinical phenotype can be further refined into functionally meaningful subprofiles [1, 2]. One such characteristic are emotional features [1, 3].

Emotional features relevant to ADHD include correlates or manifestations of difficulties with emotion processing and regulation, such as emotional impulsivity, intensity, lability, and variability [3, 4] as well as precursors of emotion processing and regulation, such as temperament (e.g., irritability and negative and positive surgency) [5, 6] and affective-motivational processing [7–9].

In the last 15 years, a body of work has accumulated on the association between ADHD and emotional features, establishing that ADHD is associated with differences in emotional processing and regulation, and in affected children, adolescents, and adults, with functional impairment [1, 3, 10–12]. Yet, questions remain about the specificity of the association between ADHD and emotional features; arguments remain that in ADHD, emotional symptoms are nothing but “old wine in new bottles”, i.e. what some call difficulties with emotional processing and regulation are nothing more than a comorbidity manifestation –e.g., anxiety, depression, or especially oppositional defiant disorder (ODD) [6, 10]. One approach to determining the extent to which in ADHD, differences in emotional features are an associated or a core feature is to examine whether genetic liability for ADHD is associated with emotional features, accounting for genetic liability and symptoms of comorbidities.

The complex genetic structure of ADHD entails polygenic variation, rare structural variants, and differences at the level of epigenetics [13]. An approach to index ADHD genetic liability is via the cumulative effect of frequent genetic variants using a polygenic score (PGS) [6]. Although this technique has not been employed in ADHD to examine the full range of emotional features, a pertinent question about the association between ADHD PGSs and behavioral aspects of temperament has been addressed in pioneering work. Findings indicated that ADHD PGSs are associated with parent-rated irritability [6, 14] and sensation seeking [6], in samples of children [6] and children and adolescents [14].

In these studies, *first*, although depression PGSs (and lifetime manifest symptoms of depression in [6]) were controlled statistically, PGSs for or manifest symptoms of anxiety and ODD –as comparably relevant comorbidities–, were not. This can confound associations between ADHD PGSs and emotional features. Genetically, the association may be confounded by genetic liability for anxiety or ODD. It is unclear whether the association is independent of any shared genetic effects on ADHD and these comorbidities. Clinically, anxiety, depression, and ODD are comorbid with ADHD [15, 16]; anxiety disorders are present in 18% [17], depression in 14% [17], and ODD [15] in roughly half of children and adolescents with ADHD. As these disorders are also associated with emotional features [15, 18, 19], it can be argued that what in ADHD appears to be an emotional symptom is simply ADHD being comorbid with anxiety and/or depression and/or ODD.

*Second*, assessing emotional features by behavioral manifestation ratings of temperament captures only one aspect (behavioral) of one precursor (temperament) of the construct [10, 20]. Assessment of changes in electrocortical response, via electroencephalogram (EEG) including event-related potentials (ERPs) and event-related power spectrum desynchronization (ERD)/synchronization (ERS) capture another aspect (electrophysiological). Assessment of such changes in electrocortical response to affectively and/or motivationally salient stimuli captures another precursor (affective-motivational processing). Specifically, presentation of affectively and/or motivationally salient stimuli or tasks is followed by a sustained positivity in the ERP waveform, i.e. the late positive potential (LPP) [21, 22] and a decrease in alpha power, i.e. alpha desynchronization [23]. Experimental paradigms designed to elicit automatic reactions to stimuli probe bottom-up processes whereas those designed to elicit controlled, regulatory responses probe top-down processes. Both the LPP [24] and the alpha ERD [23] are modulated by bottom-up image content, e.g. the LPP is enhanced whereas alpha ERD is attenuated by arousing pleasant and unpleasant emotional stimuli relative to neutral stimuli. Further, both the LPP [24] and the alpha ERD [23] are modulated by top-down processes, e.g. LPP is attenuated whereas alpha ERD is enhanced by emotion regulation [24]. Beyond emotional paradigms, both the LPP and the alpha ERD can be also be elicited by motivational paradigms (e.g. punishment and reward), including guessing paradigms such as the Doors task [25–28]. In such paradigms, the LPP and alpha ERD following loss and win feedback can be conceptualized as reflecting affective-motivational processing [26–28], specifically, in case of the LPP, extended cognitive processing of the affective value of feedback stimuli [25, 29].

### Current study

Here, our general goal is to examine whether accounting for genetic and behavioral markers of comorbidities relevant to the association of ADHD with emotional features, PGSs for ADHD are associated with electrophysiological indices of affective-motivational processing in adolescents.

Specifically, the first aim was to examine whether PGSs for ADHD are associated with LPP (Aim 1). The second aim was to examine whether PGSs for ADHD are associated with fronto-centro-parietal alpha ERD (Aim 2). Across aims, we controlled for PGSs for anxiety-, depressive-, and ADHD with comorbid disruptive behavior disorders. We also controlled for severity of ADHD, internalizing, and oppositional defiant disorder symptoms. We hypothesized that greater ADHD PGSs would be associated with lower LPP amplitude and that greater ADHD PGSs would be associated with greater alpha ERD [6, 14, 24].

Evidence indicates that ADHD pharmacotherapy may affect changes in emotional features including anger, irritability, emotional dysregulation, and emotional lability [30, 31]. Evidence further shows that childhood maltreatment is associated with increased psychopathology risk [32], at least in part, through emotion dysregulation [33, 34] and that in individuals with a given psychopathology, history of maltreatment may lead to a distinct ecophenotype [35]. Accordingly, we also accounted for current ADHD pharmacotherapy and childhood maltreatment.

## Method

### General procedure

The data analyzed herein were collected during the first and second assessment sessions of the baseline and 18-month follow-up (T2) timepoints of a larger longitudinal project, the Budapest Longitudinal Study of ADHD and Externalizing Disorders (BLADS).

In the larger study, adolescents were included if they were between the ages of 14 and 17 years and excluded if they exhibited cognitive ability below the percentile rank representing an estimated full-scale IQ score of 80 on select subtests of the Wechsler Adult Intelligence Scale–Fourth Edition (WAIS-IV) or Wechsler Intelligence Scale for Children–Fourth Edition (WISC-IV) [36, 37]; met criteria for bipolar, obsessive–compulsive or psychotic disorder on the Structured Clinical Interview for DSM-5, Clinical Version (SCID-5-CV) [38]; reported a prior diagnosis of autism spectrum disorder (severity ≥ 2); current or past neurological illness; or uncorrected, impaired vision ( <50 cm).

In the first assessment session at baseline, adolescents were administered a clinical interview (SCID-5-CV) and tests of cognitive performance (WAIS/WISC-IV), followed by genetic sampling and completion of questionnaires. In the second assessment session at baseline, adolescents completed EEG measurement and questionnaires. At follow-up, in the first assessment session, adolescents completed questionnaires and in the second assessment session, an EEG measurement. Parents completed questionnaires using Psytoolkit [39, 40] and the Qualtrics software, versions June 2020–May 2023 (Qualtrics, Provo, UT).

#### Ethical approval and consent to participate

This research was conducted in adherence to the ethical standards laid down in the 1964 Declaration of Helsinki and its later amendments and approved by the National Institute of Pharmacy and Nutrition (OGYÉI/17089-8/2019). Informed consent (and assent) was obtained from all parents (and adolescents).

### Participants

In the larger project, adolescents were a community sample of participants oversampled for ADHD; at baseline, data were available for *N* = 314 youth (*M*_age_ = 15.78 years, *SD* = 1.08; 39.2% girls), 89 (28.34%) of whom met criteria for ADHD, defined as exhibiting ≥6 for youth <17 years old or ≥5 for youth ≥17 years old inattentive or hyperactive/impulsive symptoms and showing moderate impairment (rating of ≥2) in ≥3 domains of functioning on the ADHD Rating Scale-5 [41], see *Method/ Measures*. The sample size was determined for the larger study [42, 43].

For descriptive statistics on analytic subsamples, see Table 1.

**Table 1:** Demographic and clinical characteristics of the Aim 1 & 2 samples.

	Aim 1 samples		Aim 2 sample
	LPP gain	LPP loss	Alpha ERD
*n*	166	168	84
Age (in years), *M* ± *SD* (Visit 1, baseline)	15.75 ± 1.07	15.77 ± 1.06	15.76 ± 1.05
Age (in years), *M* ± *SD* (Visit 2, baseline)	15.83 ± 1.08	15.83 ± 1.07	15.83 ± 1.06
Age (in years), *M* ± *SD* (Visit 1, follow-up)	17.25 ± 1.07	17.26 ± 1.06	17.24 ± 1.06
Age (in years), *M* ± *SD* (Visit 2, follow-up)	17.33 ± 1.06	17.33 ± 1.06	17.30 ± 1.05
Sex	71 female / 95 male	72 female / 96 male	35 female / 49 male
IQ percentile, *M* ± *SD*	62.83 ± 20.48	62.73 ± 20.5	64.57 ± 21.33
Net household income per person (in HUF)^†^, *M* ± *SD*	172363.9 ± 89332.23	171516.3 ± 89205.67	167763.3 ± 77013.34
With ADHD^#^	39 (23.49%)	39 (23.21%)	27 (32.14%)
Medication-naїve at baseline^&^	21 (53.85%)	21 (53.85%)	16 (59.26%)
On stimulant medication at baseline^&^	6 (15.38%)	6 (15.38%)	2 (7.41%)
On nonstimulant medication at baseline^&^	3 (7.69%)	3 (7.69%)	2 (7.41%)
Took ≥24-hour medication hiatus before baseline EEG^^^	18 yes / 2 no / 7 did not indicate	18 yes / 2 no / 7 did not indicate	13 yes / 1 no / 4 did not indicate
Medication-naїve at follow-up^&^	17 (43.59%)	17 (43.59%)	12 (44.44%)
On stimulant medication at follow-up^&^	1 (2.56%)	1 (2.56%)	0 (0%)
On nonstimulant medication at follow-up^&^	9 (23.08%)	9 (23.08%)	6 (22.22%)
Took ≥24-hour medication hiatus before follow-up EEG^^^	15 yes / 2 no / 7 did not indicate	15 yes / 2 no / 7 did not indicate	10 yes / 1 no / 4 did not indicate

^†^=The 2020 Hungarian average was 147 000 HUF [101]; ^#^=ADHD diagnosis was ascertained at baseline; ^&^=of those with ADHD; ^^^=adolescents who were prescribed ADHD pharmacotherapy in the past but discontinued or who were prescribed ADHD pharmacotherapy at the time of participation and took a ≥24-hour medication washout before EEG.

### Measures

#### Genotyping

Genomic DNA (isolated from saliva samples) were genotyped using the Illumina Infinium Global Screening Array-24 v3.0 BeadChip by LIFE & BRAIN GmbH (Bonn, Germany). For description of and details on quality control and imputation, see Supporting Information.

#### Electrophysiology

##### EEG paradigm

In the larger project, the Doors task [44] was applied to probe initial response to attainment of reward. Consistent with prior work [26–28], the post-feedback processing portion of the task was conceptualized as probing affective-motivational processing, specifically, extended cognitive processing of the affective value of feedback stimuli [25]. Participants completed 120 trials, divided into two blocks of 60 trials each. Each block contained 30 trials per condition (win or loss), with two conditions in total (60 trials per condition overall). Adolescents were told that in each trial, they could either lose 50 (HUF) or win 100 (HUF). Each trial began with presentation of a fixation mark (+) for 900 ms, followed by presentation of an image of two doors for 3000 ms. Adolescents were to choose one of the two doors by pressing either the number 7 for the left door or the number 8 for the right door on the numeric keypad, using their dominant hand. Finally, following a brief delay (1100 ms with a ± 50 ms jitter), feedback was presented for 1500 ms. Loss was indexed by a red “↓” and win was indexed by a green “↑”. Duration of the intertrial interval was 2000 ms with a ± 250 ms jitter. In a single block, 30 loss and 30 win trials were presented, ordered randomly. To enhance the effectiveness of the experimental manipulation, adolescents could exchange the virtual money that they accumulated for previously chosen snacks (candy, gum, popcorn, etc.).

##### EEG data acquisition and processing

EEG data acquisition and processing has been described elsewhere [42, 45]. Briefly, continuous EEG was recorded with a 64-channel BrainAmp DC system equipped with actiCAP active electrodes (Brain Products GmbH, Gilching, Germany) and digitized at a sampling rate of 1000 Hz and 16-bit resolution at baseline and equipped with actiChamp active electrodes (Brain Products GmbH, Gilching, Germany) and digitized at a sampling rate of 1000 Hz and 32-bit resolution at follow-up. Impedances were kept under 5 kΩ, and the FCz electrode was used as online reference. One electrooculogram electrode was placed below the left eye and another lateral to the outer canthus of the right eye.

All EEG processing was conducted in MATLAB R2017a. The Maryland Analysis of Developmental EEG (MADE) pipeline – based on the EEGLAB toolbox (v.2022.0) [46] and its ADJUST (v.1.1.1) [47] and FASTER (v.1.2.4) [48] plugins – was used for EEG offline processing [49]. To facilitate preprocessing, the two continuous EEG files (for the two blocks of the Doors task) were concatenated into a single EEG file for each participant. The MADE pipeline script was modified for compatibility with our EEG data and the appropriate were set parameters for preprocessing: (1) Downsampling from 1000 to 250 Hz for faster processing. (2) Filtering downsampled data, first with a high-pass filter (0.1 Hz), then with a low-pass filter (30 Hz). Based on the EEGLAB FIRfilt plugin (v.2.4), both filters were zero-phase Hamming-windowed sinc finite impulse response (FIR) filters. (3) Automatic detection and removal of bad channels by MADE using the FASTER plugin. (4) Independent component analysis (ICA) to decompose the signal and, employing a logistic infomax ICA algorithm [50], to identify artificial ICA components (blinks, electromyography, and eye movements) and, using a modified version of the ADJUST algorithm, to detect and remove artificial components. (5) Segmentation of the ICA-cleaned data into epochs with a fixed length (from −200 ms to 3000 ms around events). (6) Baseline correction of extracted trials using the 200 ms time interval prior to event onset (only for ERP but not for ERS analysis). (7) Application of a voltage threshold-based ( ± 100 μV) artifact rejection algorithm onto the baseline corrected (and uncorrected) trials to remove remaining artifacts [49]. (8) Interpolation of channels removed in step (3) using spherical spline interpolation. (9) Re-referencing of the data to the average of the electrodes located at the left and right mastoids (TP9 and TP10, respectively).

##### ERP analysis

Following MADE preprocessing, ERP averages were calculated for each condition, for each participant as follows. Mean values were calculated first across electrodes (following adult [51] and child [21, 22] studies, at CP1, CPz, CP2, P1, Pz, P2, and POz), and then across trials; average ERP waveforms were calculated in given time windows (1600–2200 ms after onset of feedback stimulus [22, 52]). As a final step, for each component, grand average ERP waveforms were calculated from individual ERP averages. Amplitude values for LPP to loss and win for each participant were used in statistical analyses. Loss and win trials achieved acceptable internal consistency by the ~tenth trial, see Figure S1.

##### ERD analysis

After applying MADE preprocessing, ERD was calculated using the MATLAB EEGLAB toolbox [46], consistent with convention in the literature [53, 54]. Time-frequency analysis was performed on 3200 ms long epochs extracted in the −200 to 3000 ms latency range around task events. Each epoch underwent convolution with a set of Morlet wavelets, which varied linearly between 2 and 7 cycles and covered frequencies from 1 to 30 Hz in 0.5 Hz steps. Power spectrum was calculated for a fronto-centro-parietal region, across F1, Fz, F2, FC1, Fz, FC2, C1, Cz, C2, CP1, CPz, CP2, P1, Pz, P2, and POz [55–57]. Baseline correction was applied using resting-state EEG data for each participant, obtained during a 2 × 3-minute resting state paradigm, for which participants were instructed to look at a fixation cross and allow their thoughts to freely wander, while their head was placed on a chin rest. Specifically, we randomly segmented the resting-state EEG recordings (collected from the same electrodes used in the Doors task) into 3200 ms epochs, same length as for the task. Then, time-frequency power values were calculated on these resting-state epochs with the same parameters as for task. We then computed the mean resting-state time-frequency power of each participant and electrode and applied it to baseline-correct the task-related EEG time-frequency power. Next, we averaged the baseline-corrected power spectrum values across the above-mentioned electrodes and calculated the extended alpha band (5–14 Hz) power within the 1600–2200 ms time window. This method allowed us to quantify desynchronization or synchronization as an average decrease or increase in power spectrum values from the resting state to the task, reflecting affective-motivational processing. The resulting desynchronization values were expressed in decibels (dB). Alpha power ERD values were used in statistical analyses.

#### Rating scale measures

The parent-reported ADHD Rating Scale-5 (ARS-5) [41] was used to assess ADHD; the Internalizing problems subscale of the Youth Self-Report 11-18 (YSR) [58] to measure anxiety and depression; the parent-reported Disruptive Behaviour Disorders Rating Scale (DBD-RS) [59] to assess ODD; and the self-reported Child Abuse and Trauma Scale (CATS) [60] was used to measure childhood maltreatment.

Prior findings indicate acceptable psychometric properties for the ARS-5 [41–43], the YSR [43, 58], the DBD-RS [43, 59], and the CATS [34]. In the entire sample, all measures exhibited at least acceptable internal consistency. For details, see Supporting Information.

### Analytic plan

All analyses were conducted in RStudio (version 2024.04.2. Build 764, R version 4.4.1.). For packages used, see Table S1.

#### PGS

PGSs were calculated with PRSice-2 (v2.3.5) [61] using publicly available GWAS summary statistics for ADHD [62], ADHD with DBD comorbidity [63], anxiety [64] and depression [65]. Variants with INFO scores <0.9 were excluded from the discovery sample before clumping (window size 250 kb, *p*-value threshold 1, *R*^2^ threshold 0.1). ADHD PGSs were calculated using target phenotypes. Adolescents were classified as with ADHD if they exhibited ≥6 (youth <17 years old) or ≥5 (youth ≥17 years old) ADHD inattentive (IA) or hyperactive/impulsive (H/I) symptoms and showed moderate impairment (rating of ≥2) in ≥3 domains of functioning on the ARS-5. Control adolescents did not meet these thresholds. Adolescents were classified as with ADHD and DBD if they also exhibited ≥4 symptoms of ODD (“probable ODD”) or ≥3 symptoms of conduct disorder (“probable conduct disorder”) on the DBD-RS. Control adolescents did not meet these thresholds.

For PGS models, *p*-value thresholds were assessed at intervals of 5e-05 between 5e-08 and 1 for the inclusion of variants. Anxiety and depressive disorder PGSs were calculated using all clumped variants.

Details on both discovery and target samples are presented in Table 2.

**Table 2:** Descriptive statistics of the discovery and target samples with polygenic score models.

Phenotype	GWAS discovery sample				Target sample						
					Target phenotype		Number of variants				
	Cases	Controls	*h*^2^_SNP_ (*SE*)	Ref	Cases	Controls	in both datasets	after clumping	in best fitting model	Variant inclusion threshold (*p*-value)	*R*^2^
ADHD	38,691	186,843	0.14 (0.01)	[62]	79	207	5,237,388	205,518	6,956	0.00400005	5.27%
ADHD with DBD	3802	31,305	0.25 (0.03)	[63]	48	184	4,893,306	139,387	116,785	0.6279	3.24%
Anxiety disorders	74,973 (28,392 proxy)	400,243 (146,771 proxy)	0.079 (0.004)	[64]	–^†^	–^†^	5,901,087	276,772	276,772	1	–
Depression	294,322	741,438	0.070 (0.002)^#^	[65]	–^†^	–^†^	5,634,359	275,791	275,791	1	–

^†^ Due to the relatively low proportion of youth meeting diagnostic criteria for either anxiety disorders or depression, instead of using target phenotypes for variant selection, PGS calculations were based on all clumped variants; ^#^ this estimate corresponds to the primary meta-analysis involving 371,184 cases and 978,703 controls; *ADHD* Attention-deficit/hyperactivity disorder, *DBD* disruptive behavior disorders, *GWAS* genome-wide association study, *h2* SNP Estimated SNP-based heritability, *R2* variance explained in the target phenotype by the best fitting polygenic score model, *SE* standard error.

#### Statistical analyses

##### Regression analysis

Across models, linear regression analyses were conducted. Independent variables of non-interest were current ADHD, internalizing, and ODD severity, current ADHD pharmacotherapy, anxiety PGSs, depression PGSs, ADHD with comorbid DBD PGSs, childhood maltreatment, as well as the first four genetic principal components and baseline values of the outcome variables. The independent variable of interest was standardized ADHD PGSs and dependent variables were T2 values of LPP to win, LPP to lose, alpha ERD. *p-*values corresponding to the independent variable of interest were adjusted for false discovery rate (FDR) [66].

Across models, distribution of residuals was checked applying the Anderson-Darling and Lilliefors-corrected Kolmogorov-Smirnov tests, homoscedasticity applying the studentized Breusch-Pagan test, and multicollinearity was checked via variance inflation factors. Where applicable, we also inspected diagnostic plots (e.g., density plots, histograms, Q-Q plots, residuals vs fitted values). In cases of model assumption violations, robust linear regression analyses were conducted using the robustbase package, and tuning parameters were set using the “KS2014” setting [67]. Multicollinearity was never severe (VIFs < 2.23).

To ensure that the inclusion of all covariates was not driving findings of significance, blocks of conceptually related covariates were added separately to each model in sensitivity analyses [68] conducted following identical steps as in main analyses (see Supporting Information).

To determine whether attrition was at random, binary logistic regression analyses were conducted in the entire (*N* = 314) sample with age, sex, ADHD status, cognitive ability, and socioeconomic status (net household income per person) as independent variables entered simultaneously and whether an adolescent had T2 data as the dependent variable.

## Results

### Attrition

The model for attrition analysis was nonsignificant: *χ*^2^(6) = 11.592, *p* = 0.072.

### Descriptives

For distribution of ADHD PGSs by ADHD diagnosis, see Figure S2.

In the entire (*n* = 284) sample, in a robust regression model with ADHD PGSs and the first four ADHD genetic principal components, χ^2^(5) = 14.759, *p* = 0.011, adj. *R*^2^ = 0.033, standardized ADHD PGSs were associated with ADHD severity, *b* = 2.404, *SE* = 0.783, *p* = 0.002.

### ERPs and ERD

**Fig. 1:**
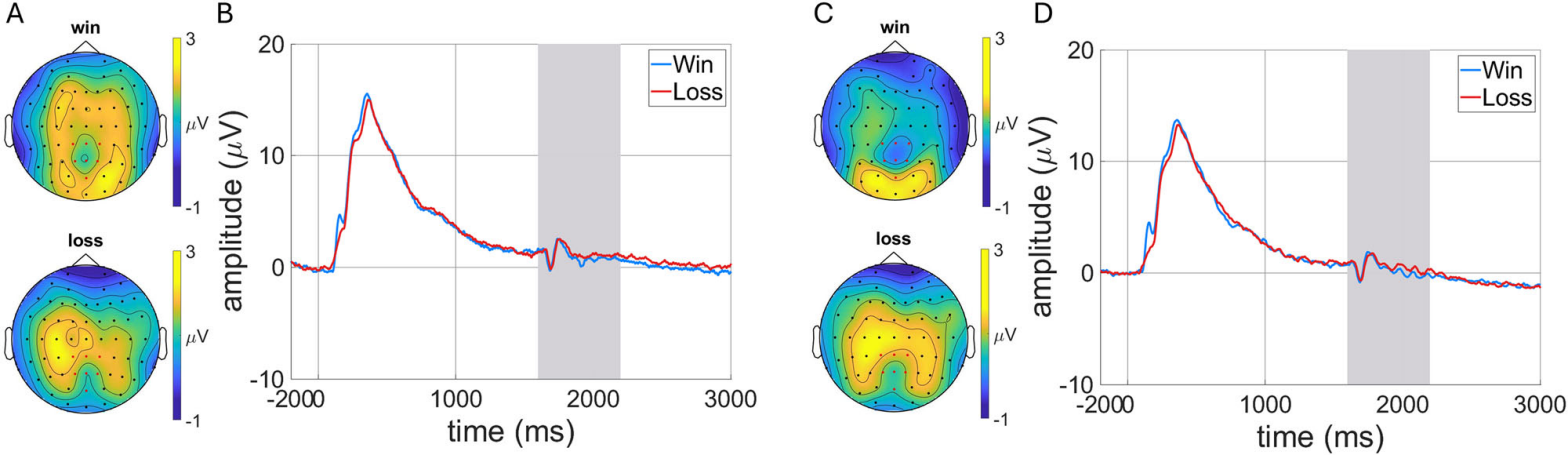
LPP to win and loss. **A** Baseline scalp distributions depicting activation to win (LPP to win) and lose (LPP to lose) in the 1600-2200 ms time window, with electrodes selected for scoring the LPP (CP1, Cpz, Cp2, P1, Pz, P2, and Poz) in red. **B** Baseline ERP grand average waveforms of the win (blue) and lose (red) condition cues. ERPs were scored in the 1600-2200 ms time window indicated by grey shading. (**C**) Follow-up scalp distributions depicting activation to win (LPP to win) and lose (LPP to lose) in the 1600-2200 ms time window, with electrodes selected for scoring the LPP (CP1, Cpz, Cp2, P1, Pz, P2, and Poz) in red. (**D**) Follow-up ERP grand average waveforms of the win (blue) and lose (red) condition cues. ERPs were scored in the 1600-2200 ms time window indicated by grey shading.

**Fig. 2:**
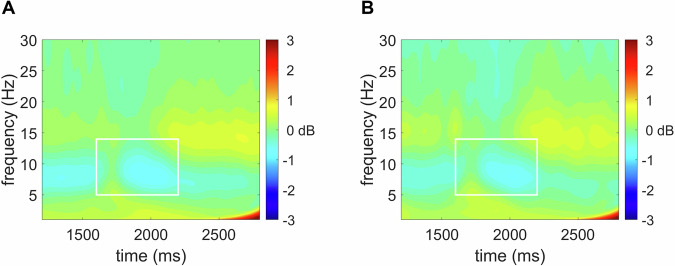
Event-related spectral perturbation of fronto-centro-parietal EEG power. Figure depicts event-related desynchronization of fronto-centro-parietal EEG power in the 1000-3000 ms post-feedback time window (scored at F1, Fz, F2, FC1, Fz, FC2, C1, Cz, C2, CP1, CPz, CP2, P1, Pz, P2, and POz), with the average of alpha calculated in the 5-14 Hz frequency range, in the 1600-2200 ms time window) for adolescents at **A** baseline and **B** 18-month follow-up.

For baseline and T2 scalp distributions and ERP grand average waveforms, see Fig. 1 and for baseline and T2 alpha ERD, see Fig. 2.

**Fig. 3:**
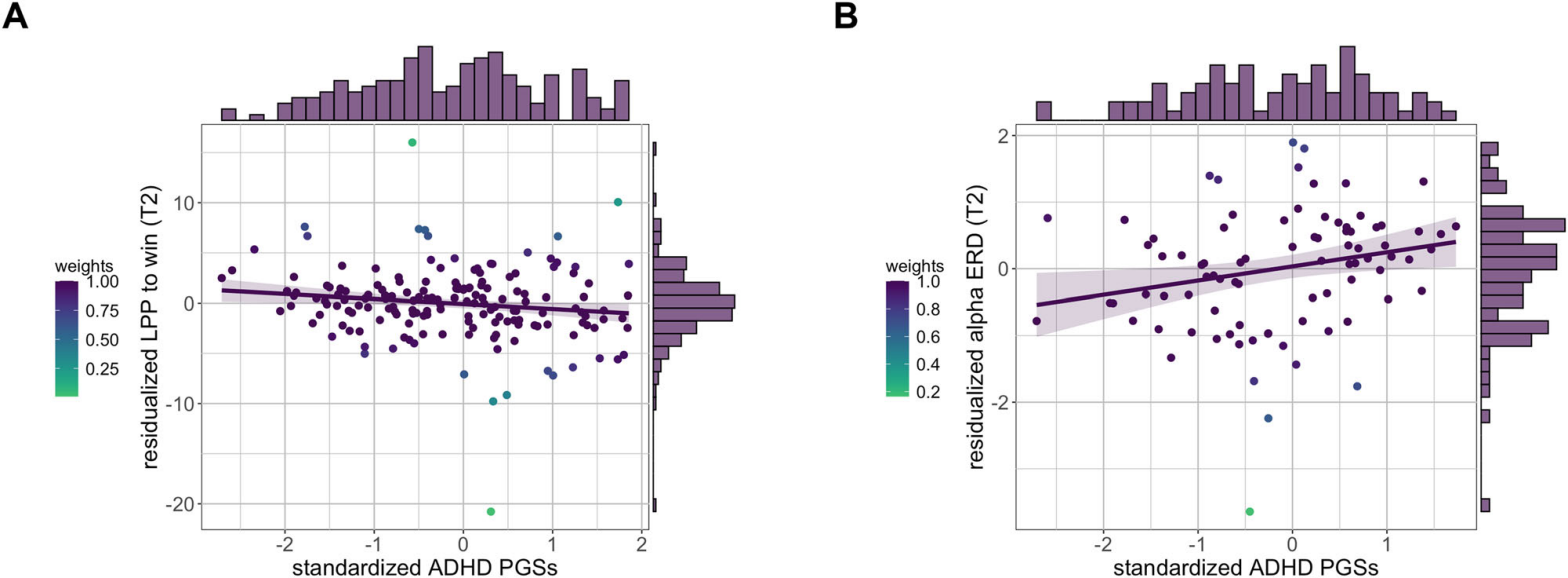
In adolescents, ADHD PGSs are associated with electrophysiological affective-motivational processing. **A** ADHD PGSs are associated, prospectively, with LPP to win. **B** ADHD PGSs are associated, prospectively, with fronto-centro-parietal alpha ERD. *Note*. Residualized LPP scores are created by regressing all covariates (current ADHD, internalizing, and ODD severity, current ADHD pharmacotherapy, childhood maltreatment, anxiety PGSs, depression PGSs, ADHD with comorbid DBD PGSs, as well as the first four genetic principal components and baseline LPP to win values) onto follow-up LPP to win values. Residualized ERD scores are created by regressing all covariates (current ADHD, internalizing, and ODD severity, current ADHD pharmacotherapy, childhood maltreatment, anxiety PGSs, depression PGSs, ADHD with comorbid DBD PGSs, as well as the first four genetic principal components and baseline alpha ERD values) onto follow-up ERD values. Data points are weighted using the analysis weights obtained from the robust regression model presented in the text.

The robust regression model predicted LPP to win, χ^2^(14) = 34.651, *p* = 0.002, adj. *R*^2^ = 0.125 (Table 3), with a negative association of standardized ADHD PGSs (*b* = −0.563, *SE* = 0.248, *p*_corr_ = 0.039) and a positive association of baseline LPP to win (*b* = 0.273, *SE* = 0.062, *p* < 0.001) and PC1 (*b* = 9.599, *SE* = 3.579, *p* = 0.008) with follow-up LPP to win (Fig. 3a). In sensitivity analyses, alternative models with adjusted covariates were comparable to main models (see Supporting Information and Figure S4).

**Table 3:** Parameter estimates for robust regression model predicting LPP to win.

	*b*	SE	*t*	*p*	95% CI		VIF
(Intercept)	−0.296	0.557	−0.531	0.596	−1.397	0.805	–
Baseline LPP to win	0.273	0.062	4.386	<0.001	0.150	0.397	1.081
Standardized ADHD PGSs	−0.563	0.248	−2.267	0.025	−1.053	−0.072	1.140
Genetic PC1	9.599	3.579	2.682	0.008	2.528	16.670	1.064
Genetic PC2	0.061	4.429	0.014	0.989	−8.691	8.812	1.070
Genetic PC3	5.118	4.061	1.260	0.210	−2.907	13.142	1.113
Genetic PC4	3.794	4.442	0.854	0.394	−4.983	12.571	1.103
Standardized anxiety disorders PGSs	−0.240	0.289	−0.830	0.408	−0.811	0.331	1.319
Standardized ADHD + DBD PGSs	0.062	0.266	0.232	0.817	−0.464	0.587	1.211
Standardized depression PGSs	0.111	0.286	0.389	0.698	−0.454	0.676	1.353
Childhood maltreatment	0.003	0.020	0.127	0.899	−0.037	0.042	1.555
ADHD medication^#^	0.141	1.046	0.135	0.893	−1.925	2.207	1.216
ADHD severity^#^	−0.016	0.030	−0.524	0.601	−0.074	0.043	2.227
ODD severity^#^	0.067	0.061	1.101	0.273	−0.053	0.187	2.187
Internalizing severity^#^	0.004	0.032	0.117	0.907	−0.060	0.067	1.344

^#^ at baseline; *ADHD* attention-deficit/hyperactivity disorder, *DBD* disruptive behavior disorders, *ODD* oppositional defiant disorder, *PC* principal component, *PGS* polygenic score, *VIF* variance inflation factor.

The linear regression model did not predict LPP to loss, *F*(14, 153) = 1.503, *p* = 0.116.

The robust regression model predicted FCP alpha ERD, χ^2^(14) = 53.160, *p* < 0.001, adj. *R*^2^ = 0.316 (Table 4), with a positive association of standardized ADHD PGSs (*b* = 0.259, *SE* = 0.114, *p*_corr_ = 0.039), of baseline ERD (*b* = 0.515, *SE* = 0.117, *p* < 0.001), and of PC2 (*b* = 4.685, *SE* = 1.409, *p* = 0.001) with follow-up ERD (Fig. 3b). In sensitivity analyses, alternative models with adjusted covariates and alpha ERD scored at 8-13 Hz were comparable to main models (see Supporting Information and Figure S4).

**Table 4:** Parameter estimates for robust regression model predicting alpha ERD.

	*b*	SE	*t*	*p*	95% CI		VIF
(Intercept)	−0.595	0.253	−2.346	0.022	−1.100	−0.089	–
Baseline alpha ERD	0.515	0.117	4.390	<0.001	0.281	0.749	1.101
Standardized ADHD PGSs	0.259	0.114	2.279	0.026	0.032	0.486	1.223
Genetic PC1	−0.879	1.271	−0.692	0.491	−3.414	1.655	1.151
Genetic PC2	4.685	1.409	3.326	0.001	1.874	7.495	1.154
Genetic PC3	−2.027	1.700	−1.192	0.237	−5.418	1.364	1.264
Genetic PC4	−0.184	1.788	−0.103	0.918	−3.752	3.383	1.261
Standardized anxiety disorders PGSs	−0.227	0.128	−1.767	0.082	−0.483	0.029	1.372
Standardized ADHD + DBD PGSs	−0.125	0.119	−1.055	0.295	−0.362	0.112	1.329
Standardized depression PGSs	0.148	0.133	1.110	0.271	−0.118	0.414	1.304
Childhood maltreatment	0.003	0.008	0.411	0.683	−0.013	0.019	1.568
ADHD medication^#^	0.177	0.433	0.408	0.685	−0.688	1.041	1.157
ADHD severity^#^	0.021	0.012	1.801	0.076	−0.002	0.045	1.979
ODD severity^#^	0.011	0.022	0.490	0.626	−0.033	0.055	2.014
Internalizing severity^#^	0.010	0.014	0.686	0.495	−0.019	0.039	1.507

^#^ at baseline; *ADHD* attention-deficit/hyperactivity disorder, *DBD* disruptive behavior disorders, *ODD* oppositional defiant disorder, *PC* principal component, *PGS* polygenic score, *VIF* variance inflation factor.

## Discussion

Historically, in ADHD, emotional features were considered a core feature. In the first clinical descriptions of ADHD by Melchior Adam Weikard in 1775, then by Alexander Crichton in 1798 and by George Still in 1902 [69], difficulties with attention and distractibility were accompanied by –and described as being *etiologically related to*– difficulties with *emotional control and stability*, manifesting in *extreme passionateness* and *morbid exaggeration of emotional excitability* [70]. In later descriptions of ADHD in the 1960s, difficulties with regulating activity, attention, and impulses and with perseveration were accompanied by *emotional excitability or lability*. However, arguably because emotional features were difficult to measure, beginning with the second DSM in 1968, the taxonomic doctrine for ADHD has been that difficulties with regulating activity, attention, and impulses are core features of ADHD whereas emotional symptoms are merely an associated feature of the disorder [70].

Given that ADHD is heterogeneous [2], “core feature” indicates that even if emotional features are part of the ADHD clinical phenotype, those are essential or manifest only in a subpopulation. Accordingly, when we ask whether emotional features should be considered “core features”, we are asking whether ADHD should be conceptualized as including difficulties not only with regulating activity, attention, and impulses but also with regulating and processing emotions.

It is against this backdrop that here, we assumed that if blunted affective-motivational processing is a core feature of ADHD, then it will be associated with ADHD genetic liability. But, if blunted affective-motivational processing is merely an associated feature of ADHD that is a comorbidity manifestation, then accounting for relevant behavioral and genetic variables, it will not be associated with ADHD genetic liability. To examine this hypothesis, we aimed to determine the extent to which accounting for relevant behavioral and genetic variables, prospectively measured electrophysiological affective-motivational processing is associated with ADHD PGSs.

Specifically, we hypothesized that greater ADHD PGSs would be associated with lower LPP amplitude and that greater ADHD PGSs would be associated with greater alpha ERD [6, 14, 24]. We found that greater ADHD PGSs were associated with an attenuated occipito-parietal LPP and an enhanced fronto-centro-parietal alpha ERD, consistent with less electrophysiological engagement in –or response to– affective-motivational processing [23]. Because associations between variables substantially vary as a function of which covariates are included in models [71], we conducted sensitivity analyses with blocks of conceptually relevant covariates entered separately. The original results were replicated with previously tested (depression PGSs and internalizing symptom severity) and without biological (genetic and pharmacotherapy variables) covariates.

Conceptually, in combination with earlier findings, these results support a conceptualization of emotional features being a core ADHD feature. Specifically, accounting for cognitive [11], comorbid [11, 72, 73], and demographic (e.g. age and sex) [72–74] characteristics, earlier findings show that (1) different aspects of emotional features (e.g. emotional dysregulation, emotional lability, temperament), assessed by different measurement modalities (e.g. behavioral [75, 76], experiential [73] and neurophysiological [77, 78]) *are phenotypically associated with ADHD*. (2) In ADHD, differences in emotional features *explain heterogeneity* [1, 10, 12] *and exacerbate* [73] *or explain functional impairment* [72, 74]. Accounting for genetic and behavioral markers of comorbidities, the current (and earlier [6, 14]) results show that (3) different aspects of emotional features –temperament and affective-motivational processing– *are associated with genetic liability for ADHD*. Taken together, emotional features are relevant to the manifestation of ADHD, prognosis in ADHD, and the etiology of ADHD.

Clinically, diagnostic procedures ideally entail a comprehensive assessment of characteristics implicated in the phenotypic presentation and prognosis of a disorder and are informed by what is known about the etiology of that disorder. Intervention planning, in turn, is ideally informed by the data obtained in the context of such diagnostic procedures. Prior findings underscore the relevance of emotional features as a characteristic implicated in the phenotypic presentation and prognosis of ADHD and the current findings underscore the relevance of affective-motivational processing as a characteristic related to the etiology of ADHD. As such, considering emotional symptoms in assessments of ADHD stands to enhance the effectiveness and precision of both diagnostic and intervention planning processes [12]. Future studies may focus on establishing the clinical and incremental utility of assessing emotional features in evaluations for ADHD, for diagnostic and intervention planning purposes [79]. Assessments could target differences in emotional features including emotional impulsivity, intensity, lability and variability [3, 4] as well as temperament (e.g., irritability and negative and positive surgency) [5, 6] and affective-motivational processing [7–9]. Techniques directly and explicitly targeting affective-motivational processing or other emotional features are currently not incorporated into treatments that are evidence-based for children and adolescents with ADHD. The need for the development of such techniques (or treatments incorporating such techniques) has been underscored in recent reviews of the treatment evidence-base [80].

Prior work has shown that across children and adolescents, ADHD PGSs are associated with parent-rated domains of temperament, accounting for depression polygenic scores [6, 14].

1. In assessing electrophysiological indices of affective-motivational processing during a reward task, we captured a new aspect of emotional features. Parent ratings of temperament, despite their advantages including administration and cost effectiveness, are inherently subjective and not informative about underlying biological mechanisms of characteristics of interest. Conversely, assessment of ERPs and ERD during controlled experimental paradigms yields a direct and objective measure of brain response that can inform mechanism-based assessment and intervention [81]. In support, e.g. children classified as behaviorally inhibited at age 3 have been observed to exhibit an attenuated error-related negativity at age 6 but an enhanced error-related negativity at age 9, indicating that the same manifest childhood temperament is associated with different neurobiological mechanisms across development [82] and may thus warrant different intervention targets. Nevertheless, we agree with others that empirical integration across data types is a priority [1] and call for combination of different measures in research on emotional features in ADHD.
2. In accounting for both etiologic and manifest markers of anxiety, depression and ODD, we focused on attenuating biases that could emerge from confounds given overlap in the causes and presentation of emotional symptoms across ADHD and its comorbidities. Manifestations –deficits in acceptance and reappraisal accompanied by avoidance, rumination and suppression– and mechanisms (e.g. attention biases [21]) of difficulties with emotion processing and regulation in anxiety and depression [83], may be less related to the manifestations (anger, exuberance, frustration intolerance, lability, negativity, reactivity [10, 11]), and mechanisms (autonomic inflexibility and reactivity [77, 84]) of emotional symptoms in ADHD. Conversely, the manifestations –anger, negativity, reactivity [5]– and mechanisms of difficulties with emotional processing and regulation in ODD [85] appear more strongly related to the manifestations and mechanisms of emotional symptoms in ADHD. Nevertheless, other characteristics and disorders, including callousness-unemotionality and conduct disorder are also relevant for the association of ADHD with differences in emotional features [86, 87] and are to be examined in the context of the aims of the current study in the future.

No association was observed between ADHD PGSs and LPP to loss. As there is no precedent on the association between ADHD PGSs and the LPP, the explanatory hypotheses we offer are speculative. There is some evidence that differences in affective and behavioral characteristics corresponding to a the positive valence –such as to monetary reward in a guessing task– are related to emergence of ADHD symptoms [88]. These characteristics may be relatively specific to ADHD symptoms (as opposed to the covariates in our analyses, e.g., ODD [88]). Conversely, differences in affective and behavioral characteristics corresponding to the negative valence –such as to monetary loss in a guessing task– may be general markers of pathology. As a result, covarying genetic liability for and manifest symptoms of anxiety, depression, and ODD may have contributed to us not having observed an association between ADHD PGSs and LPP to loss. Specifically, a body of work indicates ADHD is associated with high parent- and teacher report negative emotionality and self-report neuroticism [89–93], and dysregulation of negative emotions [10, 73]. Yet, it has been argued that as both negative emotionality and neuroticism are associated with a large number of disorders, they may be general markers of psychopathology [94], and as rating scale measures of negative emotionality include items reflecting anger and irritability, negative emotionality may be more closely related to the externalizing spectrum generally, and ODD specifically, than to ADHD per se [95]. Indeed, in some samples, compared to the number of children with ADHD characterized by low control or high surgency, only a very small subgroup are characterized by high negative affect [96].

### Directions for future research and limitations

Affective-motivational processing was probed in experimental paradigms and indices of this characteristic were measured via EEG. With regard to the LPP as an electrophysiological index, evidence is fairly robust that, when probed during the Doors task, it can be interpreted as reflecting extended cognitive processing of the affective value of feedback stimuli [25–27, 29]. However, with regard to alpha ERD, we cannot conclusively state that it can be interpreted without limitations as reflecting affective-motivational processing, especially in the absence of other measurement modalities in this study against which it could be validated.

For example, alpha ERD can also be interpreted as reflecting impaired attention [97] or within the framework of alpha inhibition theory, as reflecting impaired inhibition [55, 98]. However, despite being one of the most often investigated frequency bands, few studies examined alpha ERD during reward tasks and these have focused on ERD to anticipation of cue, -feedback, and -receipt of reward but none on ERD following feedback [29].

However, not unlike any other neural phenomenon [55, 99], alpha ERD likely does not reflect a specific cognitive process (e.g., attention, inhibition, memory). Rather, it reflects a basic mechanism involved in different contexts and processes [100]. A possibility is that alpha ERD reflects the basic mechanism associated with engagement of (affective-)motivational systems that underlie processing of the significance of stimuli [55]. Empirical findings are consistent with this insofar as those suggest there is affective modulation by pictures of the alpha ERD that is independent of picture size [56] and is apparent even after massive repetition of stimuli and when emotional stimuli serve as task-irrelevant distractors [55], indicating that alpha ERD reflects the engagement of affective-motivational systems [56]. Affective modulation of alpha ERD is also apparent by other types of cues that are motivationally salient (e.g., conditioned aversive stimuli, pleasant stimuli, or in anticipation of a potential threat), further underscoring an interpretation of the alpha ERD as reflecting cortical excitability associated with engagement of motivational systems [55]. Nevertheless, it will be key to validate alpha ERD during the Doors task against other measures of affective-motivational processing.

## Conclusions

Findings indicated that ADHD PGSs were associated with electrophysiological measures of affective-motivational processing, namely the LPP and alpha desynchronization, suggesting blunted neural response during affective-motivational processing and in case of the LPP specifically, extended cognitive processing of the affective value of feedback stimuli.

Convergence across models with different electrophysiological indices and with support from sensitivity testing indicates that our results are robust, and the conclusions drawn are independent of construct operationalization or model structuring. Although the current and previous, relevant results [6, 14] certainly do not close the debate about emotional features being an ADHD core or associated feature, they do indicate that such features are related to ADHD genetic liability but not to comorbidity risk or symptoms and, as such, point to those being a core feature.

## Supplementary information

Supplemental Material

Supplementary Figure 1

Supplementary Figure 2

Supplementary Figure 3

Supplementary Figure 4

## Data Availability

Data analyzed in this study are available from the Author of correspondence upon reasonable request.

## Code availability

Codes for generating results are available from the Author of correspondence upon reasonable request.

## Dedication

This work is dedicated to the memory of the late William Pelham Jr. As a doctoral student, in 2010, NB presented her ideas on emotion dysregulation and ADHD to Dr. Pelham, who challenged them, asking whether emotion dysregulation in ADHD was “nothing but old wine in new bottles.” His questioning became a catalyst, inspiring NB’s program of research—culminating in this manuscript—designed to establish a different perspective.

## References

[1] Nigg JT, Karalunas SL, Feczko E, Fair DA. Toward a revised nosology for attention-deficit/hyperactivity disorder heterogeneity. Biol Psychiatry Cogn Neurosci Neuroimaging. 2020;5:726–37. 32305325 10.1016/j.bpsc.2020.02.005 PMC7423612

[2] Nigg JT, Sibley MH, Thapar A, Karalunas SL. Development of ADHD: etiology, heterogeneity, and early life course. Annu Rev Dev Psychol. 2020;2:559–83. 34368774 10.1146/annurev-devpsych-060320-093413 PMC8336725

[3] Karalunas SL, Dude J, Figuracion M, Lane SP. Momentary dynamics implicate emotional features in the ADHD phenotype. Res Child Adolesc Psychopathol. 2024;52:1343–56. 38771497 10.1007/s10802-024-01206-9 PMC11694784

[4] Rosen PJJ, Epstein JNJN, Van Orden G. I know it when I quantify it: Ecological momentary assessment and recurrence quantification analysis of emotion dysregulation in children with ADHD. ADHD Atten Deficit Hyperact Disord. 2013;5:283–94. 10.1007/s12402-013-0101-2 PMC3714339 23338519

[5] Junghänel M, Thöne A-K, Ginsberg C, Görtz-Dorten A, Frenk F, Mücke K, et al. Irritability and emotional impulsivity as core feature of ADHD and ODD in children. J Psychopathol Behav Assess. 2022;44:679–97.

[6] Nigg JT, Karalunas SL, Gustafsson HC, Bhatt P, Ryabinin P, Mooney MA, et al. Evaluating chronic emotional dysregulation and irritability in relation to ADHD and depression genetic risk in children with ADHD. J Child Psychol Psychiatry. 2020;61:205–14. 31605387 10.1111/jcpp.13132 PMC6980250

[7] Sonuga-Barke EJS. The dual pathway model of AD/HD: an elaboration of neuro-developmental characteristics. Neurosci Biobehav Rev. 2003;27:593–604. 14624804 10.1016/j.neubiorev.2003.08.005

[8] Sonuga-Barke EJS, Bitsakou P, Thompson M. Beyond the dual pathway model: evidence for the dissociation of timing, inhibitory, and delay-related impairments in attention-deficit/hyperactivity disorder. J Am Acad Child Adolesc Psychiatry. 2010;49:345–55. 20410727 10.1016/j.jaac.2009.12.018

[9] Tegelbeckers J, Bunzeck N, Duzel E, Bonath B, Flechtner H-H, Krauel K. Altered salience processing in attention deficit hyperactivity disorder. Hum Brain Mapp. 2015;36:2049–60. 25648705 10.1002/hbm.22755 PMC4670482

[10] Bunford N, Evans SW, Wymbs F. ADHD and emotion dysregulation among children and adolescents. Clin Child Fam Psychol Rev. 2015;18:185–217. 26243645 10.1007/s10567-015-0187-5

[11] Graziano P, Garcia A. Attention-deficit hyperactivity disorder and children’s emotion dysregulation: a meta-analysis. Clin Psychol Rev. 2016;46:106–23. 27180913 10.1016/j.cpr.2016.04.011

[12] Karalunas SL, Gustafsson HC, Fair D, Musser ED, Nigg JT. Do we need an irritable subtype of ADHD? Replication and extension of a promising temperament profile approach to ADHD subtyping. Psychol Assess. 2019;31:236–47. 30359050 10.1037/pas0000664 PMC6345612

[13] Faraone SV, Larsson H. Genetics of attention deficit hyperactivity disorder. Mol Psychiatry. 2019;24:562–75. 29892054 10.1038/s41380-018-0070-0 PMC6477889

[14] Riglin L, Eyre O, Cooper M, Collishaw S, Martin J, Langley K, et al. Investigating the genetic underpinnings of early-life irritability. Transl Psychiatry. 2017;7:e1241. 28949337 10.1038/tp.2017.212 PMC5639253

[15] American Psychiatric Association. Diagnostic and Statistical Manual of Mental Disorders, Fifth Edition, Text Revision (DSM-5-TR). American Psychiatric Association; 2022.

[16] Mohammadi M-R, Zarafshan H, Khaleghi A, Ahmadi N, Hooshyari Z, Mostafavi S-A, et al. Prevalence of ADHD and its comorbidities in a population-based sample. J Atten Disord. 2021;25:1058–67. 31833803 10.1177/1087054719886372

[17] Larson K, Russ SA, Kahn RS, Halfon N. Patterns of comorbidity, functioning, and service use for US children with ADHD, 2007. Pediatrics. 2011;127:462–70. 21300675 10.1542/peds.2010-0165 PMC3065146

[18] Clear SJ, Gardner AA, Webb HJ, Zimmer-Gembeck MJ. Common and distinct correlates of depression, anxiety, and aggression: attachment and emotion regulation of sadness and anger. J Adult Dev. 2020;27:181–91.

[19] Masters MR, Zimmer-Gembeck MJ, Farrell LJ. Transactional associations between adolescents’ emotion dysregulation and symptoms of social anxiety and depression: a longitudinal study. J Early Adolesc. 2019;39:1085–109.

[20] Gross J. Handbook of emotion regulation. Guilford Publications; 2013.

[21] Bunford N, Kujawa A, Fitzgerald KD, Swain JE, Hanna GL, Koschmann E, et al. Neural reactivity to angry faces predicts treatment response in pediatric anxiety. J Abnorm Child Psychol. 2017;45:385–95. 27255517 10.1007/s10802-016-0168-2 PMC5800984

[22] Bunford N, Kujawa A, Swain JE, Fitzgerald KD, Monk CS, Phan KL. Attenuated neural reactivity to happy faces is associated with rule breaking and social problems in anxious youth. Eur Child Adolesc Psychiatry. 2017;26:215–30. 27341840 10.1007/s00787-016-0883-9

[23] Parvaz MA, Moeller SJ, Goldstein RZ, Proudfit GH. Electrocortical evidence of increased post-reappraisal neural reactivity and its link to depressive symptoms. Soc Cogn Affect Neurosci. 2015;10:78–84. 24526188 10.1093/scan/nsu027 PMC4994842

[24] Hajcak G, MacNamara A, Olvet DM. Event-related potentials, emotion, and emotion regulation: an integrative review. Dev Neuropsychol. 2010;35:129–55. 20390599 10.1080/87565640903526504

[25] Zheng Y, Shi P, Deng L, Jiang H, Zhou S. Contextual valence influences the neural dynamics of time and magnitude representation during feedback evaluation. Psychophysiology. 2023;60:e14335. 37194930 10.1111/psyp.14335

[26] Tan JXY, Liu P. Beyond the RewP: The reward feedback-elicited LPP and its potential associations with perceived stress and internalizing symptoms in late childhood. Int J Psychophysiol. 2023;193:112237. 37625596 10.1016/j.ijpsycho.2023.08.010

[27] Takács M, Tóth B, Szalárdy O, Bunford N. Theta and alpha activity are differentially associated with physiological and rating scale measures of affective processing in adolescents with but not without ADHD. Dev Psychopathol. 2024;36:1426–41. 10.1017/S0954579423000639 37357942

[28] Liu P, Tan JXY. Late positive potential elicited by monetary reward feedback predicts changes of disordered eating from age 11 to Age 12 in community‐dwelling girls. Int J Eat Disord. 2024;57:2106–16. 39004895 10.1002/eat.24253

[29] Glazer JE, Kelley NJ, Pornpattananangkul N, Mittal VA, Nusslock R. Beyond the FRN: Broadening the time-course of EEG and ERP components implicated in reward processing. Int J Psychophysiol. 2018;132:184–202. 29454641 10.1016/j.ijpsycho.2018.02.002

[30] Kondi K, Takács M, Kovács-Posta E, Szajli C, Sebők-Welker T, Réthelyi JM, et al. Emotion dysregulation in adolescents is normalized by ADHD pharmacological treatment. Borderline Personal Disord Emot Dysregulation. 2025;12:3. 10.1186/s40479-024-00268-x PMC11789310 39894810

[31] Posner J, Kass E, Hulvershorn L. Using Stimulants to Treat ADHD-Related Emotional Lability. Curr Psychiatry Rep. 2014;16:478. 25135778 10.1007/s11920-014-0478-4 PMC4243526

[32] Teicher MH, Gordon JB, Nemeroff CB. Recognizing the importance of childhood maltreatment as a critical factor in psychiatric diagnoses, treatment, research, prevention, and education. Mol Psychiatry. 2022;27:1331–8. 34737457 10.1038/s41380-021-01367-9 PMC8567985

[33] Gruhn MA, Compas BE. Effects of maltreatment on coping and emotion regulation in childhood and adolescence: a meta-analytic review. Child Abuse Negl. 2020;103:104446. 32200195 10.1016/j.chiabu.2020.104446 PMC12352122

[34] Sebők-Welker T, Posta E, Ágrez K, Rádosi A, Zubovics EA, Réthelyi MJ, et al. The association between prenatal maternal stress and adolescent affective outcomes is mediated by childhood maltreatment and adolescent behavioral inhibition system sensitivity. Child Psychiatry Hum Dev. 2024;55:1–21. 36738426 10.1007/s10578-023-01499-9 PMC11362206

[35] Staginnus M, Cornwell H, Toschi N, Oosterling M, Paradysz M, Smaragdi A, et al. Testing the ecophenotype model: cortical structure alterations in conduct disorder with versus without childhood maltreatment. Biol Psychiatry Cogn Neurosci Neuroimaging. 2023;8:609–19. 36925341 10.1016/j.bpsc.2022.12.012

[36] Wechsler D Wechsler intelligence scale for children–Fourth Edition (WISC-IV). 2003. 10.1080/09297040590951587 16306021

[37] Wechsler D. Wechsler adult intelligence scale–Fourth Edition (WAIS–IV). 2008.

[38] First MB, Williams JBW, Karg RS, Spitzer RL. User’s guide for the SCID-5-CV Structured Clinical Interview for DSM-5® disorders: Clinical version. Arlington, VA, US: American Psychiatric Publishing, Inc; 2016.

[39] Stoet G. PsyToolkit: a software package for programming psychological experiments using Linux. Behav Res Methods. 2010;42:1096–104. 21139177 10.3758/BRM.42.4.1096

[40] Stoet G. PsyToolkit: a novel web-based method for running online questionnaires and reaction-time experiments. Teach Psychol. 2017;44:24–31.

[41] DuPaul GJ, Power TJ, Anastopoulos AD, Reid R. ADHD Rating Scale—5 for Children and Adolescents: Checklists, Norms, and Clinical Interpretation. The Guilford Press; 2016.

[42] Hámori G, File B, Fiáth R, Pászthy B, Réthelyi JM, Ulbert I, et al. Adolescent ADHD and electrophysiological reward responsiveness: a machine learning approach to evaluate classification accuracy and prognosis. Psychiatry Res. 2023;323:115139. 36921508 10.1016/j.psychres.2023.115139

[43] Rádosi A, Ágrez K, Pászthy B, Réthelyi JM, Ulbert I, Bunford N. Concurrent and prospective associations of reward response with affective and alcohol problems: ADHD-related differential vulnerability. J Youth Adolesc. 2023;52:1856–72. 37270465 10.1007/s10964-023-01794-7

[44] Hajcak G, Moser JS, Holroyd CB, Simons RF. The feedback-related negativity reflects the binary evaluation of good versus bad outcomes. Biol Psychol. 2006;71:148–54. 16005561 10.1016/j.biopsycho.2005.04.001

[45] Hámori G, Rádosi A, Pászthy B, Réthelyi JM, Ulbert I, Fiáth R, et al. Reliability of reward ERPs in middle‐late adolescents using a custom and a standardized preprocessing pipeline. Psychophysiology. 2022;59:e14043. 35298041 10.1111/psyp.14043 PMC9541384

[46] Delorme A, Makeig S. EEGLAB: an open source toolbox for analysis of single-trial EEG dynamics including independent component analysis. J Neurosci Methods. 2004;134:9–21. 15102499 10.1016/j.jneumeth.2003.10.009

[47] Mognon A, Jovicich J, Bruzzone L, Buiatti M. ADJUST: An automatic EEG artifact detector based on the joint use of spatial and temporal features. Psychophysiology. 2011;48:229–40. 20636297 10.1111/j.1469-8986.2010.01061.x

[48] Nolan H, Whelan R, Reilly RB. FASTER: Fully Automated Statistical Thresholding for EEG artifact Rejection. J Neurosci Methods. 2010;192:152–62. 20654646 10.1016/j.jneumeth.2010.07.015

[49] Debnath R, Buzzell GA, Morales S, Bowers ME, Leach SC, Fox NA. The Maryland analysis of developmental EEG (MADE) pipeline. Psychophysiology. 2020;57:e13580. 32293719 10.1111/psyp.13580 PMC12758016

[50] Bell AJ, Sejnowski TJ. An information-maximization approach to blind separation and blind deconvolution. Neural Comput. 1995;7:1129–59. 7584893 10.1162/neco.1995.7.6.1129

[51] Stange JP, MacNamara A, Barnas O, Kennedy AE, Hajcak G, Phan KL, et al. Neural markers of attention to aversive pictures predict response to cognitive behavioral therapy in anxiety and depression. Biol Psychol. 2017;123:269–77. 27784617 10.1016/j.biopsycho.2016.10.009 PMC5503152

[52] Leutgeb V, Schäfer A, Köchel A, Scharmüller W, Schienle A. Psychophysiology of spider phobia in 8- to 12-year-old girls. Biol Psychol. 2010;85:424–31. 20851734 10.1016/j.biopsycho.2010.09.004

[53] Cohen MX. Analyzing Neural Time Series Data: Theory and Practice. MIT Press; 2014.

[54] Morales S, Bowers ME. Time-frequency analysis methods and their application in developmental EEG data. Dev Cogn Neurosci. 2022;54:101067. 35065418 10.1016/j.dcn.2022.101067 PMC8784307

[55] Codispoti M, De Cesarei A, Ferrari V. Alpha-band oscillations and emotion: A review of studies on picture perception. Psychophysiology. 2023;60:e14438. 37724827 10.1111/psyp.14438

[56] De Cesarei A, Codispoti M. Affective modulation of the LPP and α-ERD during picture viewing. Psychophysiology. 2011;48:1397–404. 21486291 10.1111/j.1469-8986.2011.01204.x

[57] Messerotti Benvenuti S, Buodo G, Mennella R, Dal Bò E, Palomba D. Appetitive and aversive motivation in depression: the temporal dynamics of task-elicited asymmetries in alpha oscillations. Sci Rep. 2019;9:17129. 31748518 10.1038/s41598-019-53639-8 PMC6868126

[58] Achenbach TM, Rescorla L *Manual for the ASEBA school-age forms & profiles: Child behavior checklist for ages 6-18, teacher’s report form, youth self-report*. 2001.

[59] Pelham W, Gnagy EM, Greenslade KE, Milich R. Teacher ratings of DSM-III-R symptoms for the disruptive behavior disorders. J Am Acad Child Adolesc Psychiatry. 1992;31:210–8. 1564021 10.1097/00004583-199203000-00006

[60] Sanders B, Becker-Lausen E. The measurement of psychological maltreatment: Early data on the child abuse and trauma scale. Child Abuse Negl. 1995;19:315–23. 9278731 10.1016/s0145-2134(94)00131-6

[61] Choi SW, O’Reilly PF. PRSice-2: polygenic risk score software for biobank-scale data. GigaScience. 2019;8:giz082. 31307061 10.1093/gigascience/giz082 PMC6629542

[62] Demontis D, Walters GB, Athanasiadis G, Walters R, Therrien K, Nielsen TT, et al. Genome-wide analyses of ADHD identify 27 risk loci, refine the genetic architecture and implicate several cognitive domains. Nat Genet. 2023;55:198–208. 36702997 10.1038/s41588-022-01285-8 PMC10914347

[63] Demontis D, Walters RK, Rajagopal VM, Waldman ID, Grove J, Als TD, et al. Risk variants and polygenic architecture of disruptive behavior disorders in the context of attention-deficit/hyperactivity disorder. Nat Commun. 2021;12:576. 33495439 10.1038/s41467-020-20443-2 PMC7835232

[64] Li W, Chen R, Feng L, Dang X, Liu J, Chen T, et al. Genome-wide meta-analysis, functional genomics and integrative analyses implicate new risk genes and therapeutic targets for anxiety disorders. Nat Hum Behav. 2023;8:361–79. 37945807 10.1038/s41562-023-01746-y

[65] Als TD, Kurki MI, Grove J, Voloudakis G, Therrien K, Tasanko E, et al. Depression pathophysiology, risk prediction of recurrence and comorbid psychiatric disorders using genome-wide analyses. Nat Med. 2023;29:1832–44. 37464041 10.1038/s41591-023-02352-1 PMC10839245

[66] Benjamini Y, Hochberg Y. Controlling the false discovery rate: a practical and powerful approach to multiple testing. J R Stat Soc Ser B Methodol. 1995;57:289–300.

[67] Koller M, Stahel WA. Nonsingular subsampling for regression S estimators with categorical predictors. Comput Stat. 2017;32:631–46.

[68] Dick AS, Lopez DA, Watts AL, Heeringa S, Reuter C, Bartsch H, et al. Meaningful associations in the adolescent brain cognitive development study. NeuroImage. 2021;239:118262. 34147629 10.1016/j.neuroimage.2021.118262 PMC8803401

[69] Barkley RA, Peters H. The Earliest Reference to ADHD in the Medical Literature? Melchior Adam Weikard’s Description in 1775 of “Attention Deficit” (Mangel der Aufmerksamkeit, Attentio Volubilis). J Atten Disord. 2012;16:623–30. 22323122 10.1177/1087054711432309

[70] Barkley RA. Deficient emotional self-regulation: a core component of attention-deficit/hyperactivity disorder. J ADHD Relat Disord. 2010;1:5–37.

[71] Hyatt CS, Owens MM, Crowe ML, Carter NT, Lynam DR, Miller JD. The quandary of covarying: a brief review and empirical examination of covariate use in structural neuroimaging studies on psychological variables. NeuroImage. 2020;205:116225. 31568872 10.1016/j.neuroimage.2019.116225

[72] Bunford N, Evans SW, Becker SP, Langberg JM. Attention-deficit/hyperactivity disorder and social skills in youth: a moderated mediation model of emotion dysregulation and depression. J Abnorm Child Psychol. 2015;43:283–96. 25037460 10.1007/s10802-014-9909-2 PMC6538387

[73] Bunford N, Evans SW, Langberg JM. Emotion dysregulation is associated with social impairment among young adolescents with ADHD. J Atten Disord. 2018;22:66–82. 24681899 10.1177/1087054714527793

[74] Fogleman ND, Slaughter KE, Rosen PJ, Leaberry KD, Walerius DM. Emotion regulation accounts for the relation between ADHD and peer victimization. J Child Fam Stud. 2019;28:2429–42.

[75] Maedgen JW, Carlson CL. Social functioning and emotional regulation in the attention deficit hyperactivity disorder subtypes. J Clin Child Psychol. 2000;29:30–42. 10693030 10.1207/S15374424jccp2901_4

[76] Melnick SM, Hinshaw SP. Emotion regulation and parenting in AD/HD and comparison boys: Linkages with social behaviors and peer preference. J Abnorm Child Psychol. 2000;28:73–86. 10772351 10.1023/a:1005174102794

[77] Bunford N, Evans SW, Zoccola PM, Owens JS, Flory K, Spiel CF. Correspondence between heart rate variability and emotion dysregulation in children, including children with ADHD. J Abnorm Child Psychol. 2017;45:1325–37. 28032274 10.1007/s10802-016-0257-2

[78] Monopoli WJ, Evans SW, Benson K, Allan NP, Owens JS, DuPaul GJ, et al. Assessment of a conceptually informed measure of emotion dysregulation: evidence of construct validity *vis a vis* impulsivity and internalizing symptoms in adolescents with ADHD. Int J Methods Psychiatr Res. 2020;29:1–14. 32898309 10.1002/mpr.1826 PMC7723178

[79] Karalunas SL, Fair D, Musser ED, Aykes K, Iyer SP, Nigg JT. Notice of Retraction and Replacement. Karalunas et al. Subtyping attention-deficit/hyperactivity disorder using temperament dimensions: toward biologically based nosologic criteria. JAMA Psychiatry. 2018;75:408–9. 2014;71(9):1015-1024. *JAMA Psychiatry* 29516086 10.1001/jamapsychiatry.2018.0013 PMC6743729

[80] Evans SW, Owens JS, Wymbs BT, Ray AR. Evidence-based psychosocial treatments for children and adolescents with attention deficit/hyperactivity disorder. J Clin Child Adolesc Psychol. 2018;47:157–98. 29257898 10.1080/15374416.2017.1390757

[81] Hajcak G, Klawohn J, Meyer A. The utility of event-related potentials in clinical psychology. Annu Rev Clin Psychol. 2019;15:71–95. 31067414 10.1146/annurev-clinpsy-050718-095457

[82] Meyer A, Hajcak G, Torpey-Newman D, Kujawa A, Olino TM, Dyson M, et al. Early temperamental fearfulness and the developmental trajectory of error-related brain activity. Dev Psychobiol. 2018;60:224–31. 29344944 10.1002/dev.21605 PMC5815917

[83] Schäfer JÖ, Naumann E, Holmes EA, Tuschen-Caffier B, Samson AC. Emotion regulation strategies in depressive and anxiety symptoms in youth: a meta-analytic review. J Youth Adolesc. 2017;46:261–76. 27734198 10.1007/s10964-016-0585-0

[84] Musser ED, Backs RW, Schmitt CF, Ablow JC, Measelle JR, Nigg JT. Emotion regulation via the autonomic nervous system in children with attention-deficit/hyperactivity disorder (ADHD). J Abnorm Child Psychol. 2011;39:841–52. 21394506 10.1007/s10802-011-9499-1 PMC3112468

[85] Leaberry KD, Rosen PJ, Fogleman ND, Walerius DM, Slaughter KE. Physiological emotion regulation in children with ADHD with and without comorbid internalizing disorders: a preliminary study. J Psychopathol Behav Assess. 2018;40:452–64.

[86] Koppány D, Hámori G, Réthelyi JM, Bunford N The Association Between Adolescent Adhd and Callous-unemotional Traits is Moderated by Electrophysiological Reinforcement Sensitivity. 2024. 10.21203/rs.3.rs-4236701/v1.

[87] Waxmonsky J, Fosco W, Waschbusch D, Babinski D, Baweja R, Pegg S, et al. The impact of irritability and callous unemotional traits on reward positivity in youth with ADHD and conduct problems. Res Child Adolesc Psychopathol. 2022;50:1027–40. 35182261 10.1007/s10802-022-00901-9 PMC9388699

[88] Bunford N, Kujawa A, Dyson M, Olino T, Klein DN. Examination of developmental pathways from preschool temperament to early adolescent ADHD symptoms through initial responsiveness to reward. Dev Psychopathol. 2022;34:841–53. 33722319 10.1017/S0954579420002199

[89] Nigg JT, John OP, Blaskey LG, Huang-Pollock CL, Willcutt EG, Hinshaw SP, et al. Big five dimensions and ADHD symptoms: links between personality traits and clinical symptoms. J Pers Soc Psychol. 2002;83:451–69. 12150240 10.1037/0022-3514.83.2.451

[90] Martel MM, Nigg JT. Child ADHD and personality/temperament traits of reactive and effortful control, resiliency, and emotionality. J Child Psychol Psychiatry. 2006;47:1175–83. 17076757 10.1111/j.1469-7610.2006.01629.x

[91] Singh AL, Waldman ID. The etiology of associations between negative emotionality and childhood externalizing disorders. J Abnorm Psychol. 2010;119:376–88. 20455610 10.1037/a0019342

[92] White JD. Personality, temperament and ADHD: a review of the literature. Personal Individ Differ. 1999;27:589–98.

[93] Healey DM, Marks DJ, Halperin JM. Examining the interplay among negative emotionality, cognitive functioning, and attention deficit/hyperactivity disorder symptom severity. J Int Neuropsychol Soc. 2011;17:502–10. 21466739 10.1017/S1355617711000294

[94] Lahey BB. Public health significance of neuroticism. Am Psychol. 2009;64:241–56. 19449983 10.1037/a0015309 PMC2792076

[95] Nigg JT, Goldsmith HH, Sachek J. Temperament and attention deficit hyperactivity disorder: the development of a multiple pathway model. J Clin Child Adolesc Psychol. 2004;33:42–53. 15028540 10.1207/S15374424JCCP3301_5

[96] Martel MM. Dispositional trait types of ADHD in young children. J Atten Disord. 2016;20:43–52. 23239785 10.1177/1087054712466915 PMC4308572

[97] Deiber M-P, Hasler R, Colin J, Dayer A, Aubry J-M, Baggio S, et al. Linking alpha oscillations, attention and inhibitory control in adult ADHD with EEG neurofeedback. NeuroImage Clin. 2020;25:102145. 31911342 10.1016/j.nicl.2019.102145 PMC6948256

[98] Jensen O. Distractor inhibition by alpha oscillations is controlled by an indirect mechanism governed by goal-relevant information. Commun Psychol. 2024;2:1–11. 38665356 10.1038/s44271-024-00081-w PMC11041682

[99] Cacioppo JT, Tassinary LG. Inferring psychological significance from physiological signals. Am Psychol. 1990;45:16–28. 2297166 10.1037//0003-066x.45.1.16

[100] Klimesch W. Alpha-band oscillations, attention, and controlled access to stored information. Trends Cogn Sci. 2012;16:606–17. 23141428 10.1016/j.tics.2012.10.007 PMC3507158

[101] 2021. https://www.ksh.hu/docs/hun/xftp/idoszaki/hazteletszinv/2020/index.html Központi Statisztikai Hivatal. A háztartások életszínvonala, 2020. Gyorstáj. Keresetek 2021 Márc.

