# Supplementary material for: Not just old wine in new bottles: Polygenic liability for ADHD is associated with electrophysiological affective-motivational processing beyond anxiety, depression, and ODD
Source: Transl Psychiatry. 2025 Jun 24;15:213. doi: 10.1038/s41398-025-03434-z (PMC12187935; doi:10.1038/s41398-025-03434-z)
Supplement: Supplementary file 1 — Supplemental Material [file 41398_2025_3434_MOESM1_ESM.docx]

SUPPORTING INFORMATION TO:

Not just old wine in new bottles:

Polygenic liability for ADHD is associated with electrophysiological affective-motivational processing beyond anxiety, depression, and ODD

**Supplementary Methods**

**Measures**

***ADHD severity.*** The ARS-5^1^ is a 30-item parent- and teacher-report measure of the past 6-month presence and severity of DSM-5 ADHD symptoms and functional impairment in children (5-10 years) and adolescents (11—17 years). The ARS-5 measures ADHD symptoms through inattention, hyperactivity/impulsivity, and ARS-5 Total scales: 9 inattentive symptom items and 9 hyperactivity/impulsivity symptom items. The ARS-5 measures impairment across six domains: relationship with significant others (family members for the home version), relationship with peers, academic functioning, behavioral functioning, homework performance and self-esteem (2×6 impairment items, with one set corresponding to inattention and one to hyperactivity/impulsivity). Parents and teachers rate items on a four-point scale: 0 - ‘never or rarely’ to 4 - ‘very often’ in case of symptoms and 0 - ‘no problem’ to 3 - ‘severe problem’ in case of impairment. Higher scores indicate more severe symptoms and more severe impairment. Prior findings indicate both the original (e.g., internal consistency and 6-week test-retest reliability; factor structure; concurrent validity and predictive validity) ^1^ and the Hungarian translation (internal consistency)^2,3^ have acceptable psychometric properties. In the entire (*N*=314) sample, the adolescent home (i.e., parent-report) version of the ARS-5 Total scale exhibited acceptable internal consistency at baseline (α=.953; ω*_t_*=.954) and was used in analyses.

***Internalizing problems.*** The Youth Self-Report 11-18^4^ is a 112-item self-report questionnaire of adaptive behavior and functioning in adolescents (ages 11-18). The Youth Self-Report measures adaptive behavior and functioning through *competence scales*: academic performance, activities, and social competence; and impaired functioning via *DSM-oriented scales*: anxiety problems, depressive problems, somatic problems, attention-deficit/ hyperactivity problems, oppositional defiant problems, and conduct problems; as well as *syndrome scales*: anxious/depressed, depressed/withdrawn, somatic complaints, attention problems, social problems, thought problems, aggressive behavior, rule-breaking behavior, externalizing problems and internalizing problems. Adolescents rate items on a 3-point scale (0 - ‘Not True’, 1 - ‘Somewhat or Sometimes True’, 2 - ‘Very True or often True’). Higher scores indicate more severe symptoms and more severe impairment.

Prior findings indicate both the original (i.e. internal consistency and 8-16-day test-retest reliability)^4^ and the Hungarian translation (convergent validity)^3^ have acceptable psychometric properties. In the entire sample (*n*=295), the Internalizing Problems subscale exhibited acceptable internal consistency at baseline (α=.913; ω*_t_*=.915) and was used in analyses.

***ODD severity.*** The Disruptive Behaviour Disorders Rating Scale^5^ is a 45-item parent- and teacher-report measure of the presence and severity of DSM-III-R ADHD symptoms (9 inattentive symptom items and 9 hyperactivity/impulsivity symptom items), ODD (8 items), and CD symptoms (15 items) in children and adolescents (with an additional 4 behavioral difficulty items). Parents and teachers rate items on a four-point scale (0 - ‘not at all’ to 3 - ‘very much’). Higher scores indicate more severe symptoms. Prior findings indicate both the original (i.e. internal consistency and factor structure)^5–8^ and the Hungarian translation (convergent validity)^3^ have acceptable psychometric properties. In the entire (*N*=314) sample, the parent-report version ODD subscale exhibited acceptable internal consistency at baseline (α=.912; ω*_t_*=.913) and was used in analyses. Because items reflect DSM-III-R symptom wording, those were modified to match DSM-5 symptom wording ^9^.

***Child Abuse and Trauma Scale (CATS)***^10^. The CATS is a 38-item self-report measure of stress and/ or trauma in the childhood home, in adolescents and adults. The CATS is comprised of 3 subscales: *Neglect/Negative Home Atmosphere* (assessing loneliness and general stress at home)*,* Punishment (assessing severe, strict, or irrational punishment) and *Sexual Abuse* (assessing direct and indirect sexual abuse). Adolescents and adults rate the frequency with which they experienced each event on a 5-point Likert-type response format scale (0 - ‘Never’ to 4 - ‘Always’), basing their ratings on the behavior of whichever parent executed the described behavior more frequently/ severely^10^. Prior findings indicate both the original (i.e. internal consistency) (Sanders, 1995) and the Hungarian translation (i.e. internal consistency)^11,12^ exhibited acceptable psychometric properties. In the entire (*N*=314) sample, the CATS Total scale exhibited acceptable internal consistency at baseline (α=.915; ω*_t_*=.918) and was used in analyses.

**Genetic analysis**

Genotyping array data were available for 333 individuals and 745,980 variants.

***Quality Control (QC)*.** Before QC, copy number variants, insertions-deletions and pseudo-autosomal regions were removed. *n*=12 participants were excluded to ensure independence, as some participants were related.

Pre-imputation QC was conducted with PLINK (v1.90b7.2)^13^ and custom scripts^14^. As recommended by Marees et al. (2018), using a liberal, >20% missingness rate threshold first, all variants not meeting this criterion were excluded, then the same exclusion was performed at the level of individuals. This was followed by the same two steps applying a stricter missingness threshold of >1%. Samples were checked for a discrepancy between genotype-based vs self-reported sex. Non-autosomal variants, as well as those with a minor allele frequency <1% were removed. Deviations from the Hardy-Weinberg equilibrium was assessed at *p*<1e-6. After linkage disequilibrium (LD)-pruning (window size 1500kb, step size 150 variants, pairwise *r*^2^ threshold .2), samples were checked for excessive heterozygosity defined as deviating from the sample mean by at least three standard deviations. On the same LD-pruned dataset, data was checked for signs of cryptic relatedness using a pi-hat threshold of .1875.

Strands were aligned to the 1000 Genomes Phase 3^16^ reference panel with Genotype Harmonizer (v1.4.25)^17^ using default parameters, with the exception of not excluding unreferenced variants. Pre-imputation checks were performed with the HRC-1000G-check-bim-v4.3.0 script (<https://www.chg.ox.ac.uk/~wrayner/tools/>) using the Haplotype Reference Consortium^18^ reference panel, version r1.1, as recommended in the Trans-Omics for Precision Medicine (TOPMed) Imputation Server’s documentation (<https://topmedimpute.readthedocs.io/en/latest/prepare-your-data/>). Genomic coordinates were converted from GRCh37/hg19 to GRCh38/hg38 using triple-liftOver^19^. Finally, PLINK binaries were converted to the VCF format using BCFtools (v1.19)^20^.

At the end of pre-imputation QC, data were available for 306 individuals and 400,960 variants in total.

***Imputation*.** Data were imputed using the TOPMed Imputation Server^21^. 97.27% of the genotyped data overlapped with the TOPMed r3 reference panel, apps@topmed-r3@1.0.0 (hg38), with 389,289 variants passing on-server QC. Data were phased with EAGLE (v2.4)^22^ using the Haplotype Reference Consortium^18^ reference panel, then imputed with Minimac4 (v2.0.0-beta2)^21^ using the TOPMed r3 reference panel.

Non-biallelic variants, as well as those with imputation quality *r*^2^<.8 were removed using BCFtools. VCF files converted back to PLINK binaries using PLINK (v2.00a5.10LM AVX2 AMD)^13^, and genotype probabilities were converted to hard calls using a best-guess genotype certainty of .9. Duplicated variants and positions, variants without an rsID, or with a minor allele frequency <1% were excluded. As all four GWAS summary statistics were on the GRCh37/hg19 build, genomic coordinates were converted back from the GRCh38/hg38 build using triple-liftOver.

Following post-imputation QC, 7,351,006 variants were available for polygenic score (PGS) analysis in total.

***Population substructure*.** Allele frequencies may vary as a function of genetic ancestry and may induce spurious correlations or attenuate true associations unless statistically controlled for. Even with a relatively homogeneous sample such as the current sample, subtle differences in genetic ancestry may still bias statistical analyses, therefore, to assess population substructure, principal component analysis was conducted on 302,812 LD-pruned variants from the imputed, QC’d dataset using the same parameters for LD-pruning as for previous steps. Based on the Scree plot (Figure S3), four of the ten extracted components were retained for use as covariates in all statistical analyses. These components explained 10.66%, 10.50%, 9.94% and 9.88% variance, respectively. Pairwise scatterplots were examined between these four components, which indicated no apparent population stratification but highlighted the presence of a handful of potential outliers.

**Supplementary Results**

**Sensitivity analyses of alternative models with previously tested covariates**

The robust regression model with previously tested variables^23^, i.e. depression PGSs and internalizing severity (but without anxiety and ADHD with DBD comorbidity PGSs and ODD severity) predicted LPP to win, χ^2^(10)=38.201, *p*<.001, adj. *R*^2^=.142, with a negative association of standardized ADHD PGSs (*b*=-.493, *SE*=.234, *p*=.037) and a positive association of baseline LPP to win (*b*=.296, *SE*=.056, *p*<.001) and PC1 (*b*=7.684, *SE*=3.583, *p*=.033) with follow-up LPP to win (Table S2 and Figure S4).

The robust regression model with depression PGSs and internalizing severity (but without anxiety and ADHD with DBD comorbidity PGSs and ODD severity) did not predict LPP to lose, χ^2^(10)=11.126, *p*=.348.

The robust regression model with depression PGSs and internalizing severity (but without anxiety disorders and ADHD with DBD comorbidity PGSs and ODD severity) predicted fronto-centro-parietal alpha ERD, χ^2^(10)=50.730, *p*<.001, adj. *R*^2^=.316, with a positive association of standardized ADHD PGSs (*b*=.208, *SE*=.100, *p*=.041), of baseline ERD (*b*=.481, *SE*=.105, *p*<.001), of PC2 (*b*=4.174, *SE*=1.271, *p=*.002), and of ADHD severity (*b*=.021, *SE*=.008, *p=*.013) with follow-up ERD (Table S3 and Figure S4).

**Sensitivity analyses of alternative models excluding biological covariates**

The robust regression model without ADHD pharmacotherapy, anxiety PGSs, depression PGSs, and ADHD with DBD comorbidity PGSs, predicted LPP to win, χ^2^(10)=34.289, *p*<.001, adj. *R*^2^=.149, with a negative association of standardized ADHD PGSs (*b*=-.566, *SE*=.236, *p*=.018) and a positive association of baseline LPP to win (*b*=.272, *SE*=.060, *p*<.001) and of PC1 (*b*=9.617, *SE*=3.479, *p*=.006) with follow-up LPP to win (Table S4 and Figure S4).

The regression model without ADHD pharmacotherapy, anxiety PGSs, depression PGSs, and ADHD with DBD comorbidity PGSs, did not predict LPP to lose, *F*(10, 157)=1.723, *p*=.080.

The regression model without ADHD pharmacotherapy, anxiety PGSs, depression PGSs, and ADHD with DBD comorbidity PGSs, predicted fronto-centro-parietal alpha ERD, *F*(10, 73)=4.103, *p*<.001, adj. *R*^2^=.272, with a positive association of baseline ERD (*b*=.504, *SE*=.120, *p*<.001) and of PC2 (*b*=4.030, *SE*=1.423, *p*=.006), but a non-significant association of standardized ADHD PGSs (*b*=.227, *SE*=.115, *p*=.052) with follow-up ERD (Table S5 and Figure S4).

**Sensitivity analyses of alpha ERD scored at 8-13 Hz**

The linear regression model predicted FCP alpha ERD, (*F*(14, 69)=3.817, *p*<.001; adj *R*^2^=.322) (Table S6), with a positive association of standardized ADHD PGSs (*b*=.255, *SE*=.123, *p*=.042), of baseline ERD (*b*=.518, *SE*=.114, *p*<.001), and of PC2 (*b*=5.350, *SE*=1.528, *p*<.001) with follow-up ERD (Table S6).

**References**

1 DuPaul GJ, Power TJ, Anastopoulos AD, Reid R. *ADHD Rating Scale-5 for Children and Adolescents*. The Guilford Press: New York - London, 2016.

2 Hámori G, File B, Fiáth R, Pászthy B, Réthelyi JM, Ulbert I *et al.* Adolescent ADHD and electrophysiological reward responsiveness: A machine learning approach to evaluate classification accuracy and prognosis. *Psychiatry Research* 2023; **323**: 115139.

3 Rádosi A, Ágrez K, Pászthy B, Réthelyi JM, Ulbert I, Bunford N. Concurrent and Prospective Associations of Reward Response with Affective and Alcohol Problems: ADHD-Related Differential Vulnerability. *J Youth Adolescence* 2023; **52**: 1856–1872.

4 Achenbach T, Rescorla L. *Manual for the ASEBA School-Age Forms & Profiles*. University of Vermont, Research Center for Children, Youth, & Families: Burlington, VT, 2001.

5 Pillow DR, Pelham WE, Hoza B, Molina BSG, Stultz CH. Confirmatory factor analyses examining attention deficit hyperactivity disorder symptoms and other childhood disruptive behaviors. *Journal of Abnormal Child Psychology* 1998; **26**: 293–309.

6 Bunford N, Brandt NE, Golden C, Dykstra JB, Suhr JA, Owens JS. Attention-Deficit/Hyperactivity Disorder Symptoms Mediate the Association between Deficits in Executive Functioning and Social Impairment in Children. *Journal of Abnormal Child Psychology* 2015; **43**: 133–147.

7 Owens JS, Hoza B. Conditional probabilities of disruptive behavior disorder symptoms predicting DSM-IV ADHD subtypes and ODD. *Journal of Attention Disorders* 2003; **7**: 11–28.

8 Van Eck K, Finney SJ, Evans SW. Parent Report of ADHD Symptoms of Early Adolescents: A Confirmatory Factor Analysis of the Disruptive Behavior Disorders Scale. *Educational and Psychological Measurement* 2010; **70**: 1042–1059.

9 American Psychiatric Association. Diagnostic and Statistical Manual of Mental Disorders, 5th Edition (DSM-5). *Diagnostic and Statistical Manual of Mental Disorders 4th edition TR* 2013; : 280.

10 Sanders B, Becker-Lausen E. The measurement of psychological maltreatment: Early data on the child abuse and trauma scale. *Child Abuse and Neglect* 1995; **19**: 315–323.

11 Láng A, Lénárd K. The relation between memories of childhood psychological maltreatment and Machiavellianism. *Personality and Individual Differences* 2015; **77**: 81–85.

12 Sebők-Welker T, Posta E, Ágrez K, Rádosi A, Zubovics E, Réthelyi JM *et al.* The association between prenatal maternal stress and adolescent affective outcomes is mediated by childhood maltreatment and adolescent behavioral inhibition system sensitivity. *Child psychiatry and human development* 2023. doi:10.1007/s10578-023-01499-9.

13 Chang CC, Chow CC, Tellier LC, Vattikuti S, Purcell SM, Lee JJ. Second-generation PLINK: rising to the challenge of larger and richer datasets. *GigaScience* 2015; **4**: s13742-015-0047–8.

14 Coleman JRI, Euesden J, Patel H, Folarin AA, Newhouse S, Breen G. Quality control, imputation and analysis of genome-wide genotyping data from the Illumina HumanCoreExome microarray. *Briefings in Functional Genomics* 2016; **15**: 298–304.

15 Marees AT, de Kluiver H, Stringer S, Vorspan F, Curis E, Marie-Claire C *et al.* A tutorial on conducting genome-wide association studies: Quality control and statistical analysis. *International Journal of Methods in Psychiatric Research* 2018; **27**: e1608.

16 The 1000 Genomes Project Consortium, Corresponding authors, Auton A, Abecasis GR, Steering committee, Altshuler DM *et al.* A global reference for human genetic variation. *Nature* 2015; **526**: 68–74.

17 Deelen P, Bonder M, Van Der Velde K, Westra H-J, Winder E, Hendriksen D *et al.* Genotype harmonizer: automatic strand alignment and format conversion for genotype data integration. *BMC Res Notes* 2014; **7**: 901.

18 the Haplotype Reference Consortium. A reference panel of 64,976 haplotypes for genotype imputation. *Nat Genet* 2016; **48**: 1279–1283.

19 Sheng X, Xia L, Cahoon JL, Conti DV, Haiman CA, Kachuri L *et al.* Inverted genomic regions between reference genome builds in humans impact imputation accuracy and decrease the power of association testing. *Human Genetics and Genomics Advances* 2023; **4**: 100159.

20 Danecek P, Bonfield JK, Liddle J, Marshall J, Ohan V, Pollard MO *et al.* Twelve years of SAMtools and BCFtools. *GigaScience* 2021; **10**: giab008.

21 Das S, Forer L, Schönherr S, Sidore C, Locke AE, Kwong A *et al.* Next-generation genotype imputation service and methods. *Nat Genet* 2016; **48**: 1284–1287.

22 Loh P-R, Danecek P, Palamara PF, Fuchsberger C, A Reshef Y, K Finucane H *et al.* Reference-based phasing using the Haplotype Reference Consortium panel. *Nat Genet* 2016; **48**: 1443–1448.

23 Nigg JT, Karalunas SL, Gustafsson HC, Bhatt P, Ryabinin P, Mooney MA *et al.* Evaluating chronic emotional dysregulation and irritability in relation to ADHD and depression genetic risk in children with ADHD. *Journal of Child Psychology and Psychiatry* 2020; **61**: 205–214.

24 Zeileis A, Hothorn T. Diagnostic Checking in Regression Relationships. *R News* 2002; **2**: 7–10.

25 Revelle W. psych: Procedures for Psychological, Psychometric, and Personality Research. 2024.https://CRAN.R-project.org/package=psych.

26 Fox J, Weisberg S. *An R Companion to Applied Regression*. 3rd ed. Sage: Thousand Oaks, CA, 2019https://socialsciences.mcmaster.ca/jfox/Books/Companion/.

27 Gross J, Ligges U. Five omnibus tests for testing the composite hypothesis of normality. 2015.https://CRAN.R-project.org/package=nortest.

28 Maechler M, Rousseeuw P, Croux C, Todorov V, Ruckstuhl A, Salibian-Barrera M *et al.* robustbase: Basic Robust Statistics. R package. 2023.http://robustbase.r-forge.r-project.org/ (accessed 21 Dec2023).

29 Koller M, Stahel WA. Sharpening Wald-type inference in robust regression for small samples. *Computational Statistics & Data Analysis* 2011; **55**: 2504–2515.

**Figure and table legends**

**Table S1.** *R packages employed for analyses.*

**Table S2.** *Parameter estimates for alternative robust regression model with previously tested^^^ covariates predicting LPP to win.*

*Notes. ^^^*=Reference ^23^; ^#^=at baseline; ADHD=Attention-Deficit/Hyperactivity Disorder; LPP=late positive potential; PC=principal component; PGS=polygenic score; VIF=variance inflation factor.

**Table S3.** *Parameter estimates for alternative robust regression model with previously tested^^^ covariates predicting alpha ERD.*

*Notes. ^^^*=Reference ^23^; ^#^=at baseline; ADHD=Attention-Deficit/Hyperactivity Disorder; ERD=event-related desynchronization; PC=principal component; PGS=polygenic score; VIF=variance inflation factor.

**Table S4.** *Parameter estimates for alternative robust regression model excluding biological covariates predicting LPP to win.*

*Notes.* ^#^=at baseline; ADHD=Attention-Deficit/Hyperactivity Disorder; LPP=late positive potential; ODD=Oppositional Defiant Disorder; PC=principal component; PGS=polygenic score; VIF=variance inflation factor.

**Table S5.** *Parameter estimates for alternative robust regression model excluding biological covariates predicting alpha ERD.*

*Notes.* ^#^=at baseline; ADHD=Attention-Deficit/Hyperactivity Disorder; ERD=event-related desynchronization; ODD=Oppositional Defiant Disorder; PC=principal component; PGS=polygenic score; VIF=variance inflation factor.

**Table S6.** *Parameter estimates for regression model predicting alpha ERD (narrow range).*

*Notes.* ^#^=at baseline; ADHD=Attention-Deficit/Hyperactivity Disorder; DBD=Disruptive Behavior Disorders; ODD=Oppositional Defiant Disorder; PC=principal component; PGS=polygenic score; VIF=variance inflation factor.

***Figure S1.*** *Cronbach’s α for win and loss trials as a function of number of trials.*

Figure depicts the internal consistency of the win (LPP win; blue) and loss (LPP loss; red) conditions, as measured using Cronbach’s α, at (A) baseline and (B) follow-up. Threshold for an acceptable α (≥.7) is indicated with a dashed line.

***Figure S2.*** *Distribution of ADHD PGSs across adolescents with ADHD and control participants.*

Note. ADHD classification was based on parent-report on the ARS-5; adolescents were classified as with ADHD, if they exhibited ≥6 (youth <17 years old) or ≥ 5 (youth ≥17 years old) inattentive (IA) or hyperactive/impulsive (H/I) symptoms and impairment (≥2=moderate impairment) in ≥3 areas of functioning.

***Figure S3.*** *Scree plot, genetic principal component analysis.*

***Figure S4.*** In sensitivity analyses with adjusted covariates, ADHD PGSs are associated with LPP to win (A & C) and fronto-centro-parietal alpha ERD (B & D).

Residualized LPP scores are created by regressing select covariates and baseline LPP to win values as well as the first four genetic principal components onto follow-up LPP to win values: (A-B) current ADHD severity, current ADHD pharmacotherapy, depression PGSs, internalizing severity; (C-D) current ADHD, internalizing, ODD severity, childhood maltreatment. Residualized ERD scores are created by regressing select covariates and baseline alpha ERD values as well as the first four genetic principal components onto follow-up ERD values; Where applicable, data points are weighted using the analysis weights obtained from the robust regression model presented in the text.

| **Table S1** | | | |
| --- | --- | --- | --- |
| *R packages employed for analyses.* | | | |
| statistical method/ test | R package | version number | reference |
| homoscedasticity test | lmtest | 0.9-40 | ^24^ |
| internal consistency | psych | 2.4.6.26 | ^25^ |
| multicollinearity test | car | 3.1-2 | ^26^ |
| normality tests | nortest | 1.0-4 | ^27^ |
| robust linear regression analysis | robustbase | 0.99-1 | ^28,29^ |

| **Table S2** | | | | | | | |
| --- | --- | --- | --- | --- | --- | --- | --- |
| *Parameter estimates for alternative robust regression model with previously tested^^^ covariates predicting LPP to win.* | | | | | | | |
|  | *b* | *SE* | *t* | *p* | 95%CI | | VIF |
| (Intercept) | -.282 | .480 | -.589 | .557 | -1.229 | .665 | - |
| Baseline LPP to win | .296 | .056 | 5.265 | <.001 | .185 | .407 | 1.054 |
| Standardized ADHD PGSs | -.493 | .234 | -2.106 | .037 | -.955 | -.031 | 1.074 |
| Genetic PC1 | 7.684 | 3.583 | 2.145 | .033 | .612 | 14.756 | 1.037 |
| Genetic PC2 | .735 | 4.393 | .167 | .867 | -7.937 | 9.406 | 1.032 |
| Genetic PC3 | 4.489 | 3.970 | 1.131 | .260 | -3.348 | 12.325 | 1.067 |
| Genetic PC4 | 2.682 | 4.210 | .637 | .525 | -5.626 | 10.991 | 1.085 |
| Standardized depression PGSs | .060 | .256 | .236 | .814 | -.445 | .566 | 1.121 |
| Internalizing severity^#^ | .027 | .026 | 1.023 | .308 | -.025 | .078 | 1.052 |
| ADHD medication^#^ | .339 | .935 | .363 | .717 | -1.507 | 2.185 | 1.162 |
| ADHD severity^#^ | .009 | .020 | .462 | .645 | -.031 | .050 | 1.188 |
| *Notes. ^^^*=Reference ^23^; ^#^=at baseline; ADHD=Attention-Deficit/Hyperactivity Disorder; LPP=late positive potential; PC=principal component; PGS=polygenic score; VIF=variance inflation factor. | | | | | | | |

| **Table S3** | | | | | | | |
| --- | --- | --- | --- | --- | --- | --- | --- |
| *Parameter estimates for alternative robust regression model with previously tested^^^ covariates predicting alpha ERD.* | | | | | | | |
|  | *b* | *SE* | *t* | *p* | 95%CI | | VIF |
| (Intercept) | -.431 | .209 | -2.062 | .042 | -.847 | -.015 | - |
| Baseline alpha ERD | .481 | .105 | 4.598 | <.001 | .273 | .689 | 1.067 |
| Standardized ADHD PGSs | .208 | .100 | 2.076 | .041 | .009 | .407 | 1.105 |
| Genetic PC1 | -.834 | 1.207 | -.691 | .492 | -3.237 | 1.568 | 1.126 |
| Genetic PC2 | 4.174 | 1.271 | 3.283 | .002 | 1.644 | 6.703 | 1.031 |
| Genetic PC3 | -2.608 | 1.580 | -1.650 | .103 | -5.752 | .537 | 1.196 |
| Genetic PC4 | -.147 | 1.611 | -.091 | .928 | -3.352 | 3.059 | 1.213 |
| Standardized depression PGSs | .038 | .116 | .325 | .746 | -.193 | .268 | 1.141 |
| Internalizing severity^#^ | .009 | .011 | .810 | .421 | -.013 | .031 | 1.164 |
| ADHD medication^#^ | -.189 | .340 | -.556 | .580 | -.867 | .488 | 1.161 |
| ADHD severity^#^ | .021 | .008 | 2.532 | .013 | .005 | .038 | 1.207 |
| *Notes. ^^^*=Reference ^23^; ^#^=at baseline; ADHD=Attention-Deficit/Hyperactivity Disorder; ERD=event-related desynchronization; PC=principal component; PGS=polygenic score; VIF=variance inflation factor. | | | | | | | |

| **Table S4** | | | | | | | |
| --- | --- | --- | --- | --- | --- | --- | --- |
| *Parameter estimates for alternative robust regression model excluding biological covariates predicting LPP to win.* | | | | | | | |
|  | *b* | *SE* | *t* | *p* | 95%CI | | VIF |
| (Intercept) | -.298 | .538 | -.555 | .580 | -1.361 | .764 | - |
| Baseline LPP to win | .272 | .060 | 4.540 | <.001 | .153 | .390 | 1.035 |
| Standardized ADHD PGSs | -.566 | .236 | -2.396 | .018 | -1.032 | -.099 | 1.075 |
| Genetic PC1 | 9.617 | 3.479 | 2.764 | .006 | 2.745 | 16.489 | 1.046 |
| Genetic PC2 | .062 | 4.257 | .015 | .988 | -8.348 | 8.472 | 1.033 |
| Genetic PC3 | 4.913 | 3.956 | 1.242 | .216 | -2.901 | 12.726 | 1.099 |
| Genetic PC4 | 4.670 | 4.269 | 1.094 | .276 | -3.763 | 13.103 | 1.052 |
| Childhood maltreatment | .005 | .019 | .250 | .803 | -.033 | .043 | 1.477 |
| ADHD severity^#^ | -.013 | .028 | -.473 | .637 | -.068 | .042 | 2.006 |
| ODD severity^#^ | .058 | .059 | .992 | .323 | -.058 | .174 | 2.126 |
| Internalizing severity^#^ | .002 | .031 | .076 | .939 | -.059 | .064 | 1.307 |
| *Notes.* ^#^=at baseline; ADHD=Attention-Deficit/Hyperactivity Disorder; LPP=late positive potential; ODD=Oppositional Defiant Disorder; PC=principal component; PGS=polygenic score; VIF=variance inflation factor. | | | | | | | |

| **Table S5** | | | | | | | |
| --- | --- | --- | --- | --- | --- | --- | --- |
| *Parameter estimates for alternative robust regression model excluding biological covariates predicting alpha ERD.* | | | | | | | |
|  | *b* | *SE* | *t* | *p* | 95%CI | | VIF |
| (Intercept) | -.447 | .264 | -1.696 | .094 | -.972 | .078 | - |
| baseline alpha ERD | .504 | .120 | 4.189 | <.001 | .264 | .743 | 1.043 |
| standardized ADHD PGSs | .227 | .115 | 1.975 | .052 | -.002 | .457 | 1.130 |
| Genetic PC1 | -1.344 | 1.302 | -1.032 | .305 | -3.938 | 1.251 | 1.124 |
| Genetic PC2 | 4.030 | 1.423 | 2.833 | .006 | 1.194 | 6.865 | 1.054 |
| Genetic PC3 | -2.413 | 1.771 | -1.363 | .177 | -5.942 | 1.116 | 1.254 |
| Genetic PC4 | 1.468 | 1.771 | .829 | .410 | -2.061 | 4.997 | 1.160 |
| Childhood maltreatment | .005 | .008 | .670 | .505 | -.010 | .021 | 1.415 |
| ADHD severity^#^ | .019 | .012 | 1.610 | .112 | -.004 | .042 | 1.729 |
| ODD severity^#^ | -.000 | .022 | -.017 | .987 | -.044 | .044 | 1.840 |
| Internalizing severity^#^ | .000 | .014 | .010 | .992 | -.029 | .029 | 1.415 |
| *Notes.* ^#^=at baseline; ADHD=Attention-Deficit/Hyperactivity Disorder; ERD=event-related desynchronization; ODD=Oppositional Defiant Disorder; PC=principal component; PGS=polygenic score; VIF=variance inflation factor. | | | | | | | |

| **Table S6** | | | | | | | |
| --- | --- | --- | --- | --- | --- | --- | --- |
| *Parameter estimates for regression model predicting alpha ERD (narrow range).* | | | | | | | |
|  | *b* | SE | *t* | *p* | 95% CI | | VIF |
| (Intercept) | -.441 | .274 | -1.610 | .112 | -.987 | .105 | - |
| Baseline alpha ERD | .518 | .114 | 4.539 | <.001 | .290 | .746 | 1.118 |
| Standardized ADHD PGSs | .255 | .123 | 2.068 | .042 | .009 | .501 | 1.222 |
| Genetic PC1 | -1.022 | 1.360 | -.751 | .455 | -3.735 | 1.692 | 1.154 |
| Genetic PC2 | 5.350 | 1.528 | 3.501 | .001 | 2.301 | 8.399 | 1.144 |
| Genetic PC3 | -2.096 | 1.852 | -1.132 | .262 | -5.790 | 1.598 | 1.289 |
| Genetic PC4 | 1.667 | 1.906 | .874 | .385 | -2.137 | 5.470 | 1.265 |
| Standardized anxiety disorders PGSs | -.114 | .135 | -.846 | .400 | -.384 | .155 | 1.341 |
| Standardized ADHD+DBD PGSs | -.109 | .126 | -.859 | .393 | -.361 | .143 | 1.327 |
| Standardized depression PGSs | .153 | .143 | 1.065 | .291 | -.133 | .438 | 1.339 |
| Childhood maltreatment | .005 | .009 | .591 | .557 | -.012 | .022 | 1.607 |
| ADHD medication^#^ | .016 | .458 | .035 | .972 | -.897 | .929 | 1.163 |
| ADHD severity^#^ | .018 | .013 | 1.385 | .171 | -.008 | .043 | 1.990 |
| ODD severity^#^ | .004 | .024 | .151 | .880 | -.044 | .051 | 2.009 |
| Internalizing severity^#^ | -.004 | .015 | -.274 | .785 | -.035 | .026 | 1.505 |
| *Notes.* ^#^=at baseline; ADHD=Attention-Deficit/Hyperactivity Disorder; DBD=Disruptive Behavior Disorders; ODD=Oppositional Defiant Disorder; PC=principal component; PGS=polygenic score; VIF=variance inflation factor. | | | | | | | |
