# Supplementary figures and images for: Not just old wine in new bottles: Polygenic liability for ADHD is associated with electrophysiological affective-motivational processing beyond anxiety, depression, and ODD
Source: Transl Psychiatry. 2025 Jun 24;15:213. doi: 10.1038/s41398-025-03434-z (PMC12187935; doi:10.1038/s41398-025-03434-z)

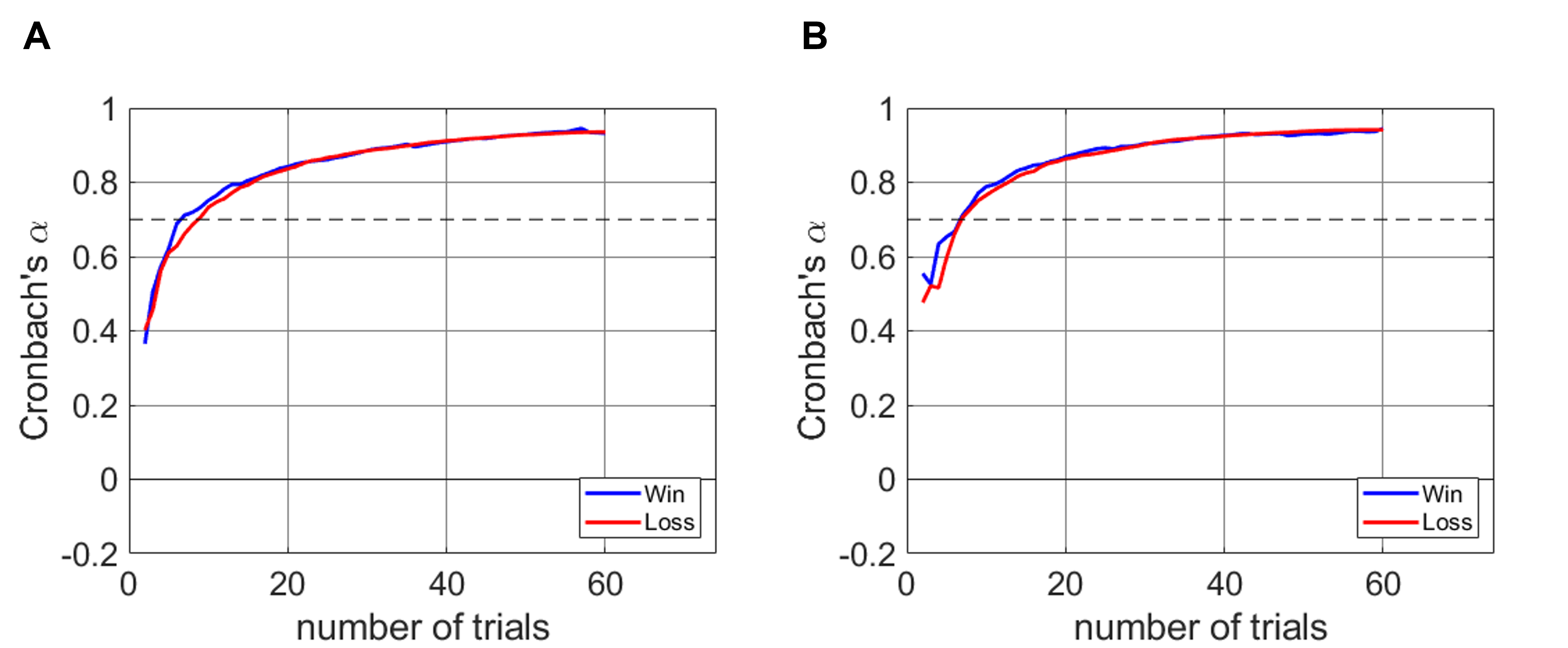

Supplement: Supplementary file 2 — Supplementary Figure 1 [file 41398_2025_3434_MOESM2_ESM.png]

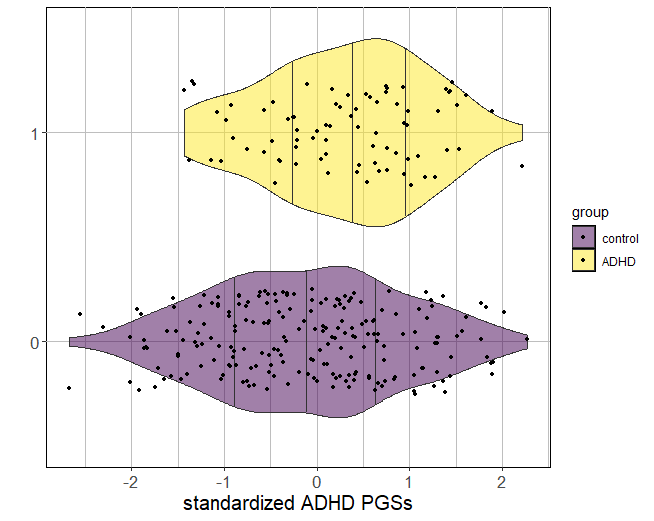

Supplement: Supplementary file 3 — Supplementary Figure 2 [file 41398_2025_3434_MOESM3_ESM.png]

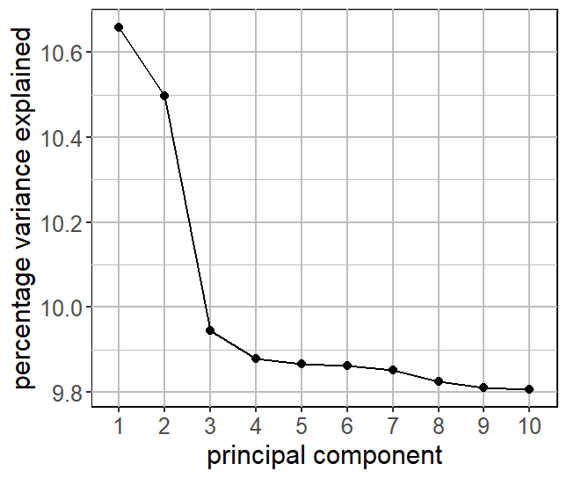

Supplement: Supplementary file 4 — Supplementary Figure 3 [file 41398_2025_3434_MOESM4_ESM.png]

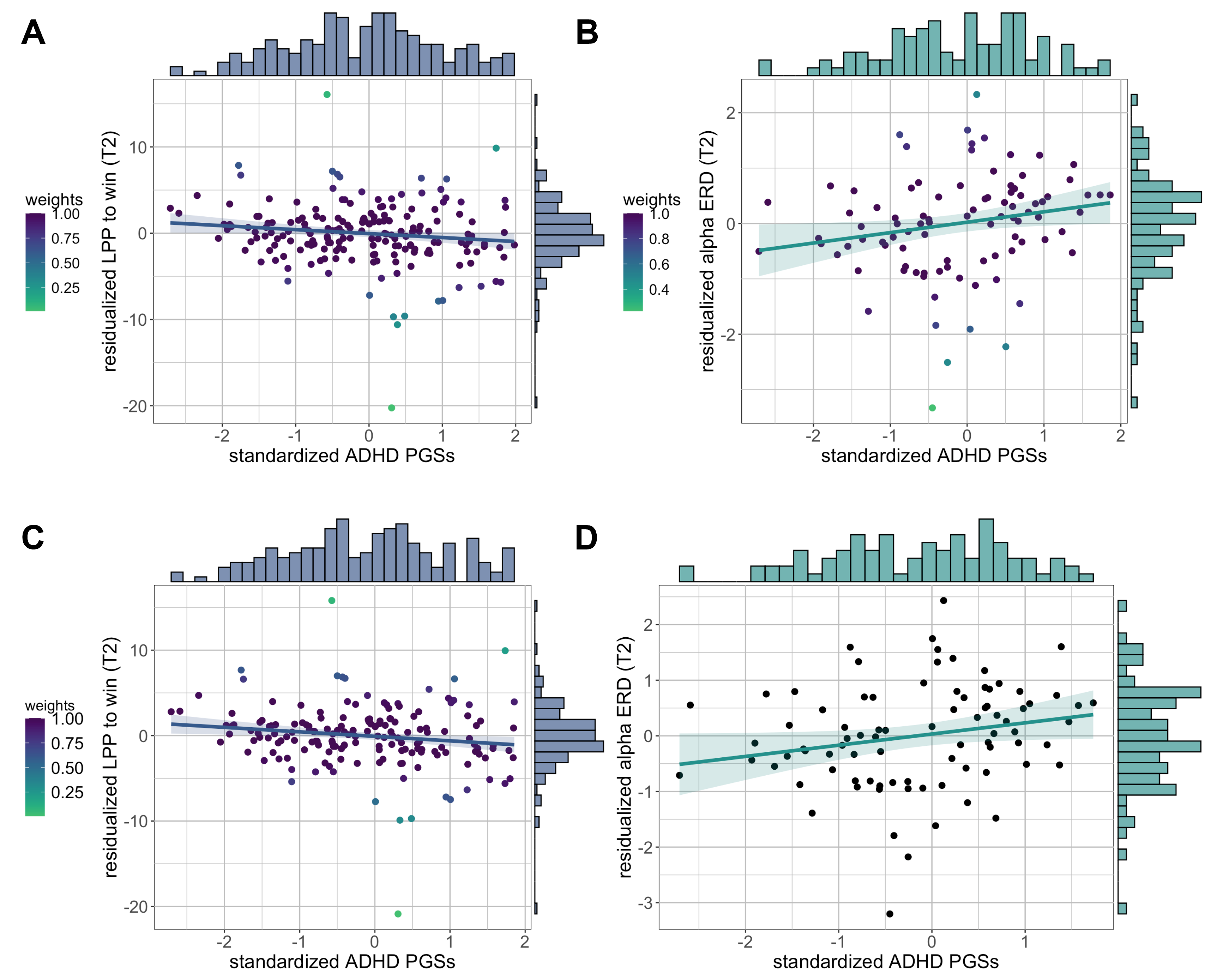

Supplement: Supplementary file 5 — Supplementary Figure 4 [file 41398_2025_3434_MOESM5_ESM.png]
